# Porous sorbents for the capture of radioactive iodine compounds: a review†

**DOI:** 10.1039/c8ra04775h

**Published:** 2018-08-17

**Authors:** Joffrey Huve, Andrey Ryzhikov, Habiba Nouali, Virginie Lalia, Grégoire Augé, T. Jean Daou

**Affiliations:** Université de Haute-Alsace, Equipe Matériaux à Porosité Contrôlée (MPC), CNRS IS2M UMR 7361 F-68100 Mulhouse France jean.daou@uha.fr +33 3 89 33 67 39; Université de Strasbourg France; ONET Technologies, 270 Chemin des Agriculteurs 26700 Pierrelatte France; ONET Technologies 13258 Marseille Cedex 9 France

## Abstract

The number of studies on the capture of radioactive iodine compounds by porous sorbents has regained major importance in the last few years. In fact, nuclear energy is facing major issues related to operational safety and the treatment and safe disposal of generated radioactive waste. In particular during nuclear accidents, such as that in 2011 at Fukushima, gaseous radionuclides have been released in the off-gas stream. Among these, radionuclides that are highly volatile and harmful to health such as long-lived ^129^I, short-lived ^131^I and organic compounds such as methyl iodide (CH_3_I) have been released. Immediate and effective means of capturing and storing these radionuclides are needed. In the present review, we focus on porous sorbents for the capture and storage of radioactive iodine compounds. Concerns with, and limitations of, the existing sorbents with respect to operating conditions and their capacities for iodine capture are discussed and compared.

## Abstract 2

In the capture of radioactive iodine compounds by porous sorbents, concerns with, and limitations of, the existing sorbents with respect to operating conditions and their capacities for iodine capture are discussed and compared.

## Graphical abstract

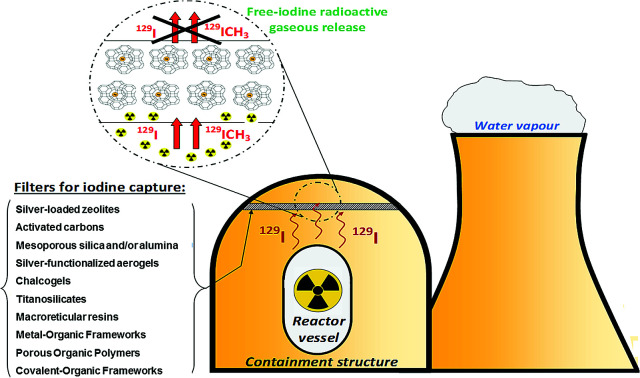

## 1. Introduction

**Fig. 1:**
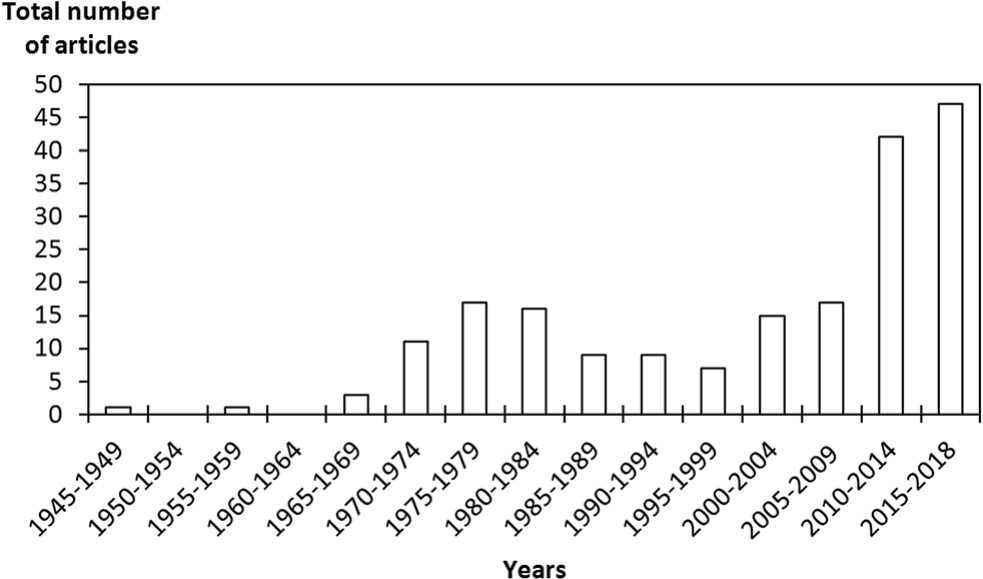
Evolution of the number of publications per year on the capture of radioactive iodine contaminants by solid porous sorbent materials.

Nuclear power plants are one of the main sources of electrical energy. Nuclear energy is safe, clean, without excessive greenhouse gas emissions and economically competitive. Although it is facing major issues related to operational safety and the treatment and safe disposal of generated radioactive waste,^1,2^ it remains an important part of today's energy mix. One of the main problems is as follows: during the aqueous reprocessing of used nuclear fuels and/or during a severe nuclear accident (Chernobyl in 1986 and Fukushima in 2011), there is a strong risk of the emission of gaseous radionuclides in the released off-gas stream.^3,4^ Gaseous radioactive wastes are a direct threat to the population and the environment owing to their easy dispersion through the atmosphere. The main components of such gaseous waste streams include the fission products technetium (^99^Tc), cesium (^137^Cs) and strontium (^90^Sr), as well as actinides, lanthanides and various volatile radionuclides (^129^I, ^131^I, ^3^H, ^14^C, ^85^Kr, *etc.*).^5–8^ Particular attention should be paid to iodine compounds, which are particularly abundant. ^129^I is a highly volatile long-lived isotope with a half-life (*t*_1/2_) of ∼1.57 × 10^7^ years, which needs to be captured and reliably stored during its long decay. In contrast, ^131^I is a volatile short-lived isotope with a *t*_1/2_ of ∼8.02 days, which needs to be captured immediately after being released, as it tends to accumulate and become concentrated in the thyroid gland, which seriously affects human metabolic processes.^9–15^ In addition, radioactive iodine is likely to form organic compounds such as methyl iodide (CH_3_I) with hydrocarbons and other volatile organic compounds present in the gas stream. Therefore, there is a strong interest in the nuclear energy community to develop alternative and effective means of capturing and storing these radionuclides. An efficient solution for iodine capture may consist of combining existing filtration devices such as wet scrubbing methods^8,16^ (Mercurex, Iodox, electrolytic and caustic scrubbing), sand bed filters and metallic filters with a supplementary capture step that potentially uses solid inorganic porous materials. Porous sorbents have been in the forefront of the removal of radioactive contaminants thanks to promising results and significant advantages such as high removal efficiency and adsorption capacities, high thermal stability, low maintenance cost, and the large range of structures and functionalizations that can be obtained.^17^ Numerous studies were carried out from the early 1960s to the late 1980s on the use of sorbents for the capture of radioactive iodine compounds with a specific focus on nuclear accidents^16–64^ (Fig. 1). These studies first focused on activated carbons and their forms impregnated with KI and/or TEDA^18,22,23,27,36,43,51,57,58^ and silver-exchanged zeolites for the capture of iodine.^17,21,29–32,34,37,38,40,47,52,56^ These sorbents were highly praised for their good performance in iodine capture and were used in industrial applications for the trapping and treatment of iodine in the off-gas stream (in particular, activated carbons owing to their very low production cost). However, the high interest in these materials suddenly disappeared during the 1990s and early 2000s, when few studies were carried out on the subject.^65–73^ Since 2010, a resurgence in studies on iodine capture has been observed (Fig. 1). Activated carbons and zeolites were re-investigated and evaluated with greater accuracy and new methods (computational screening and studies, density functional theory (DFT), modelling, diffuse-reflectance infrared Fourier transform spectroscopy (DRIFTS),…) for a better understanding of iodine capture and its performance.^74–81^ In addition, new porous sorbents have been added to the list of potential sorbents for iodine trapping, including titanosilicates,^82,83^ mesoporous silicas,^6,81,84–86^ silver-impregnated silica and alumina (Ag/SiO_2_ and Ag/Al_2_O_3_, respectively),^19,35,52,61,65,70,87–97^ silver-functionalized aerogels, chalcogels,^7,98–108^ macroreticular ion exchange resins,^16,39,41,42,44–46,50,53,55^ metal– and covalent–organic frameworks (MOFs and COFs, respectively)^6,14,109–132^ and porous organic polymers (POPs).^133–143^ In the present review, we focus essentially on porous sorbents for the capture of radioactive iodine compounds and discuss concerns and limitations with respect to operating conditions.

## 2. Capture of radioactive iodine contaminants by solid porous sorbent materials

### 2.1. Activated carbon

Activated carbons (ACs) are materials that are essentially composed of carbonaceous matter with a porous structure. Thanks to their low production cost, fairly large specific surface area (300–4000 m^2^ g^−1^), easily tuned structure and porosity (pore size ranging from 4.5 to 60 Å), as well as good adsorption capacities, activated carbons are particularly attractive for the capture of radioactive iodine compounds.^144^ Activated carbons are generally produced in two steps: firstly by carbonization (or pyrolysis), and secondly by an activation step.^144,145^ The carbonization step generates porosity within the crude carbonaceous matter by removing elements other than carbon. Afterward, an activation procedure is carried out to increase the adsorption capacity of the material. Different kinds of activation procedure can be carried out, namely, physical activation (in the presence of water vapour and air under pressure) or chemical activation (usually by treatment with phosphoric acid).^145^ Furthermore, it is possible to increase the adsorption capacities of activated carbons by a wise choice of the crude starting materials (charcoal, bamboo, coconuts and walnuts) and by adjusting the carbonization^146^ and activation steps.^145^ Iodine species such as iodine (I_2_) and methyl iodide (CH_3_I) are generally physisorbed on activated carbons, but in real conditions of utilization at a high relative humidity and high temperature (in a nuclear power plant or during a nuclear incident), the efficacity of the adsorption of such materials tends to be reduced owing to competition for adsorption sites between water and iodine species. For this reason, it is necessary to increase the affinity of activated carbons for iodine species, which can be achieved by functionalizing or impregnating the material with organic and/or inorganic compounds. The commonest compounds used for the treatment and capture of iodine compounds are triethylenediamine (TEDA)^6,18,43,69,73,147–155^ and potassium iodide (KI).^18,22,23,43,47,58,149^ Recent studies from 2010 to 2017 on the subject have essentially focused on the utilization of activated carbon impregnated with TEDA for the capture of radioactive iodine.

#### 2.1.1. Activated carbons impregnated with TEDA

**Fig. 2:**
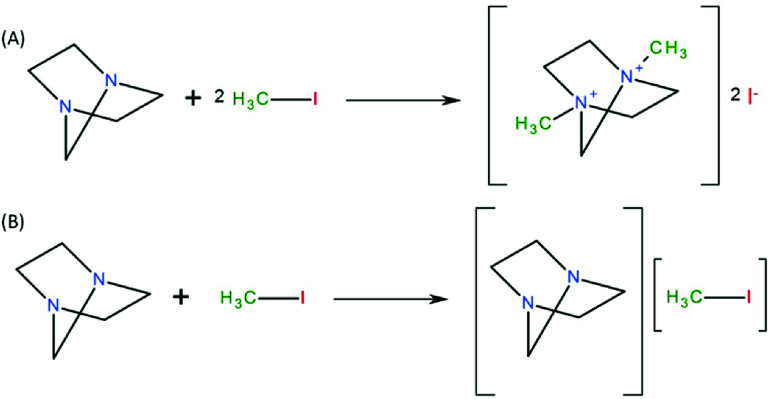
(A) Formation of a non-volatile quaternary ammonium salt by an SN_2_ mechanism between TEDA and CH_3_I. (B) Secondary neutral complex formed between TEDA and CH_3_I.^73^

In scientific studies, triethylenediamine (TEDA, C_6_H_12_N_2_), also known as 1,4-diazabicyclo[2.2.2]octane (DABCO), is the commonest impregnating agent used in activated carbon filters for the capture of radioactive iodine species. TEDA has a melting point and a boiling point of 158 °C and 174 °C, respectively, and displays strongly hygroscopic properties. To increase the iodine adsorption capacities of activated carbons, these materials are impregnated with TEDA with a concentration of usually between 0 and 10 wt%.^6,17,27,57,148,151,156,157^ However, a decrease in the accessibility of the surface and pores was observed by Chinn *et al.*^158^ and González-García *et al.*^147,157^ when the TEDA concentration was increased, which can be explained by the formation of TEDA clusters that blocked the entrances of pores. However, despite this phenomenon, it has been recognized that the presence of TEDA in activated carbons increases their iodine capture capacity (especially for CH_3_I).^147,157^ Deitz and Jonas^62^ first thought that the capture of radioactive CH_3_I by TEDA was a first-order catalytic reaction. However, according to studies by Fessenden *et al.*^159^ and Wilhelm,^57^ TEDA impregnated into activated carbons is transformed by reacting with iodine compounds such as CH_3_I *via* SN2 nucleophilic substitution into a quaternary amine.^57,58,73,159^ The general mechanism elaborated by these authors is illustrated in Fig. 2A.

**Fig. 3:**
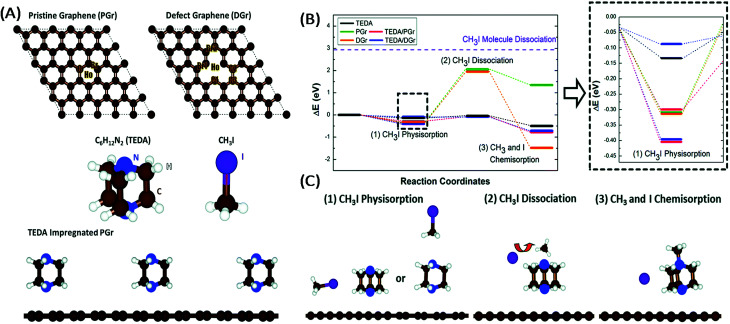
(A) Model systems of the surfaces of activated carbons (pristine graphene (PGr) and defect graphene (DGr)), the impregnating agent (TEDA), CH_3_I, H_2_O and PGr impregnated with TEDA. (B) Free energy diagram of the adsorption of CH_3_I on the surface of different systems. The dashed purple line represents the dissociation energy of a CH_3_I molecule. (C) Schematic figures for each reaction step on AC impregnated with TEDA. Reproduced in part from ref. 150 with the permission of the PCCP Owner Societies.

On the other hand, Chun *et al.*^150^ by means of energetics diagrams showed that the impregnation of TEDA into graphene helps to lower the activation energy barriers involved in the dissociation of methyl iodide (CH_3_I) into I^−^ and CH_3_ by the formation of a stable quaternary ammonium salt (Fig. 3). Kim^20^ highlighted the fact that other neutral species can form during the interaction of methyl iodide with TEDA (Fig. 2B).

Besides, the mechanism of iodine (I_2_) capture by TEDA has not been the focus of studies thus far. Nonetheless, several authors, such as Fessenden *et al.*^159^ and Takahashi *et al.*,^160^ admitted that during a severe nuclear accident radioactive iodine (I_2_) can decompose in the presence of water and/or oxygen into several iodine species (I^−^, IO_3_^−^, HOI) and can readily react with methane (CH_4_) to form CH_3_I.^161^ However, no information on the mechanism of the adsorption of these iodine species (except CH_3_I) by activated carbon or TEDA has been reported.

Various studies have been carried out to determine the performance of activated carbon impregnated with TEDA in the capture of iodine species^18,23,27,43,67,73,147^ (ESI, Table 1†). TEDA drastically increases the performance and adsorption capacities of activated carbons for iodine species at low temperatures. However, in these studies the test conditions and corresponding performance evaluations were not always representative of real conditions (temperature > 80 °C and relative humidity > 40%) that would be expected during a severe nuclear accident. Hence, major factors such as the temperature (*T*, °C) and relative humidity (R.H.%) should be taken into consideration for data analysis.

Therefore, it is crucial to study the behaviour of activated carbons impregnated with TEDA in the presence of humidity. Upon an examination of all the studies carried out on this subject, humidity was revealed to be a key factor that controls the adsorption performance of activated carbons.^66,69,147^ Nakamura *et al.*^162^ demonstrated that water molecules formed clusters that were bound in a network by hydrogen bonds to the surface of activated carbons regardless of their hydrophobicity or hydrophilicity. Furthermore, it was also observed that water filled the porous network of the activated carbons, which hindered the accessibility of active sites for iodine species. Therefore, the adsorption efficiency of these sorbents declined significantly owing to adsorption competition between water vapour and iodine species. However, the problems caused by humidity can be alleviated by the presence of TEDA in the activated carbons. In fact, methyl iodide (CH_3_I) interacts more strongly with the support.^150^ Moreover, computational studies showed that TEDA attracts water molecules (to form hydroxide ions) that are localized on the surface and in the porous network of the adsorbent, which frees the active sites needed for the adsorption of iodine species.^150^ Given that during a severe nuclear accident water is present in larger quantities than iodine species, the reaction of TEDA with water will be favoured.^159^

During a severe nuclear accident, in a case in which the cooling system would stop running, the process and/or system would eventually be subject to an increase in temperature. Therefore, it is essential to study the adsorption capacities of activated carbon impregnated with TEDA at high temperatures, taking into consideration the fact that TEDA has a melting point of 158 °C and an autoignition temperature of around 330 °C. However, it was observed several times, notably in the study by Park *et al.*^73^ on activated carbon and in the study by Ampelogova *et al.*^148^ on carbon fibres, that even at temperatures that are close to its boiling point TEDA maintains an acceptable capacity for the adsorption of methyl iodide thanks to chemisorption-type interactions (reaction of CH_3_I and TEDA to form a quaternary ammonium salt), in contrast to non-impregnated activated carbon, of which the adsorption capacity is controlled by physisorption-type interactions, although it was shown that the adsorption capacity of activated carbon impregnated with TEDA is reduced by a factor of 4 between 30 and 150 °C. The contribution of TEDA to the adsorption (chemisorption) of CH_3_I was determined to be 25.4% at 70 °C by the authors, whereas this contribution increases to 73.3% at 150 °C. These observations confirm that TEDA plays an important role in the capture of methyl iodide at high temperatures in an irreversible way (chemisorption interactions) and can be used up to a temperature of 150 °C (below the melting point of TEDA) in nuclear plants. It still needs to be confirmed that the autoignition temperature of TEDA and the thermal effect of its decomposition in the conditions of a real nuclear accident will not be detrimental to the capture performance of these materials. However, non-impregnated activated carbon loses adsorption efficiency when the temperature rises (weak physisorption interaction). Moreover, it can be noted that at low temperatures (30 °C) non-impregnated activated carbon possesses adsorption capacities that are as good as, if not even better than, those when impregnated with TEDA (Park *et al.*^67,73^ and González-García *et al.*^147^). TEDA was able to counterbalance the detrimental effect of humidity on the adsorption capacities of activated carbons by freeing the active sites of the support (water has a stronger affinity for TEDA than for the active sites).

#### 2.1.2. Activated carbons impregnated with KI

On an industrial scale, potassium iodide (KI) is an impregnating compound that is widely used with activated carbon filters for the capture of radioactive iodine. It is highly thermostable with a melting point and boiling point of 686 °C and 1330 °C, respectively. Potassium iodide improves the iodine capture performance of activated carbons *via* an increase in irreversible interactions. According to Deitz^18^ and Zhou *et al.*,^155^ the mechanism of the capture of radioactive iodine species on activated carbons impregnated with KI involves isotopic exchange between the radioactive iodine species in the gaseous phase and the stable iodine in KI. The following reversible reaction is believed to occur: CH_3_^131^I_(g)_ + K^127^I_(ads)_ ↔ CH_3_^127^I_(g)_ + K^131^I_(ads)_. Because this reaction is reversible, it is necessary to have an excess amount of non-radioactive iodine (K^127^I) to guarantee the efficient capture of radioactive iodine species. Activated carbons are generally impregnated with 0 to 10 wt% KI.^18,20,24–27,43,51,148,149,155^ Various studies, especially during the 1980s and 1990s, were carried out to determine the performance of the capture of iodine species on activated carbon impregnated with KI (ESI, Table 2†). Upon an examination of the work by Chien *et al.*,^149^ it is clearly observed that potassium iodide significantly increases the performance of activated carbon in the capture of radioactive iodine species. However, the lack of information on the test conditions used for evaluating the performance of these materials^149^ and the fact that there are no comparisons between non-impregnated and KI-impregnated activated carbon^155^ in some articles are regrettable. It should be noted that at a high concentration KI drastically limits the accessibility of the pores in the support.

As indicated in Section 2.1.1, the adsorption capacities of activated carbons are particularly affected by humidity. In comparison with TEDA, KI does not possess such a high affinity for water. In 1981, Decourcière^33^ studied the evolution of the decontamination factor (DF)‡‡The DF is defined as the ratio of the original concentration of the contaminant to the concentration that remains after removal; a DF of 10 represents a removal of radionuclides of 90%. for an activated carbon impregnated with 1 wt% KI in the presence of humidity (in the relative humidity range of 40–96%). A decrease in the decontamination factor from DF = 10^4^ to DF < 10^2^ for a relative humidity of 40% and 96%, respectively, was observed. The author concluded that the filtration system should not be used if the relative humidity is higher than 40% and should be equipped with a heating apparatus to reduce the relative humidity of air. However, during a severe nuclear accident, the relative humidity will probably exceed 40%. Therefore, it is essential that activated carbons impregnated with KI remain fully operational for the capture of iodine species even in conditions of high relative humidity. Qi-dong *et al.*^25^ reached the same conclusion with an activated carbon impregnated with 2 wt% KI + 2 wt% TEDA. Kitani *et al.*^43^ and Deuber *et al.*^27^ achieved adsorption efficiencies§§The adsorption efficiency (*E*%) is defined as the fraction of iodine captured by the material and can be related to the decontamination factor by the equation *E* = (1 − [1/DF]) × 100. of 97% and 99%, respectively, at a relative humidity of >90%. In conclusion, thus far it has been difficult to estimate whether KI is directly affected by humidity or the loss of performance is caused only by blocking of the pores and the active sites of the activated carbons. Nevertheless, humidity is detrimental for activated carbons impregnated with KI, which is why a combination of KI and TEDA is generally (or widely) used. Kitani *et al.*^43^ studied the influence of temperature on activated carbons impregnated with 5 to 10 wt% KI at a relative humidity of 90%. A decrease in the efficiency of iodine adsorption with temperature from 97.8% at 25 °C (5 wt% KI) to 93.6% at 70 °C (despite an increase in concentration to 10 wt% KI for the latter value) was observed. We could argue that this slight decrease in adsorption efficiency may be due to a decline in the accessibility of the active sites in the activated carbon caused by the increase in the KI loading rather than the increase in temperature. For activated carbons impregnated with KI + TEDA,^25^ the decontamination factor (DF) decreases by a factor of 10^2^ as the temperature rises from 40 °C to 70 °C. Therefore, it is clear that the temperature plays a major role in the iodine capture performance of activated carbons impregnated with KI.

#### 2.1.3. Limitations of impregnated activated carbons

There are several problematic issues with the use of activated carbons for the capture of radioactive volatile iodine species other than alterations at high temperatures and in the presence of humidity. Firstly, it is generally accepted in the scientific community that the aging of activated carbons is perfectly capable of altering their adsorption performance.^25–27,148^ Deuber^26^ studied the performance of activated carbons impregnated with TEDA and/or KI over a time period of 0 to 12 months at 30 °C and 130 °C with a relative humidity of 95% and 2%, respectively, for various bed lengths. It is clear from the penetration profiles that were obtained that the iodine adsorption performance was affected over time. In the case of adsorbents impregnated with TEDA, one can observe an increase in the penetration of iodine species through a bed with a length of 25 cm by a factor of 10^2^ at 30 °C and a factor of 10^2^ to 10^1^ at 130 °C after 12 months. Similar results were obtained in the case of adsorbents impregnated with KI. Therefore, the aging of activated carbons can become a major issue in the long term, especially if a severe nuclear accident occurs at the end of life of this material. According to Jubin,^16^ even though activated carbons are preferentially used in the nuclear field, especially for the capture of radioactive iodine, these materials cannot be good candidates for several reasons: they are characterized by a low autoignition temperature and in contact with other compounds, such as NO_*x*_, their iodine adsorption capacities are reduced and the formation of explosive compounds is likely.

### 2.2. Ag-exchanged zeolites

Zeolites are crystalline microporous solids that belong to the group of aluminosilicates and the subgroup of tectosilicates. Their structure is composed of a three-dimensional arrangement of tetrahedral TO_4_ units (T = Si, Al, *etc.*). The presence of pores, channels, molecule-sized cavities and charge-compensating cations inside these materials make their properties particularly interesting for various applications, such as adsorption, catalysis and cation exchange.^163–168^ In the particular case of the capture of iodine species, the adsorption properties and cation exchange capacities of zeolites make them interesting and remarkable candidates for this application. In the literature, several zeolites of different structural types have been studied for the capture of iodine species, such as mordenite (MOR structural type),^29,34,74,76,77,79,80,169–178^ NaX and NaY zeolites (FAU type),^5,60,75,77,78,88,132,179^ ZSM-5 (MFI type),^74,77^ ferrierite (FER type) and zeolite beta (*BEA type)^74,77^ (ESI, Table 3†).

#### 2.2.1. Mechanisms of iodine adsorption

Two thermodynamically viable iodine-containing precipitates are likely to be formed in silver-exchanged zeolites, namely, silver iodide (AgI) and silver iodate (AgIO_3_).^37^ The formation of the silver iodide species (AgI) was reported in various studies,^21,29,34,38,40,47,52,180^ whereas Patil *et al.*^37^ showed the formation of a silver iodate precipitate (AgIO_3_). At quite elevated temperatures (>190 °C), the latter precipitate decomposed to form silver iodide (more stable at higher temperatures, eqn (1)). Scheele and Burger^29^ identified the various reactions that could potentially be triggered between iodine species (I_2_, CH_3_I) and active silver adsorption sites in silver-exchanged mordenite Ag^0^MOR (in which silver possesses different oxidation states, namely, a charged Ag^+^ form and/or a reduced Ag^0^ form). They highlighted the fact that most of these reactions are thermodynamically favourable for the capture of I_2_ and CH_3_I by mordenite-type zeolites loaded with silver (AgMOR). The iodine species formed during these reactions are mainly AgI and AgIO_3_ (eqn (2) to (8)):1
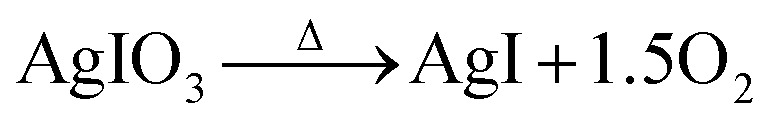

2 Ag^0^ + 0.5I_2_ ↔ AgI

3 2Ag^0^ + 2CH_3_I ↔ 2AgI + C_2_H_6_

4 Ag^0^ + CH_3_I + H_2_O ↔ AgI + CH_3_OH + 0.5H_2_

5 Ag^+^ + CH_3_I + H_2_O ↔ AgI + CH_3_OH + H^+^

6 2Ag^0^ + I_2_ + 1.5O_2_ ↔ AgI + AgIO_3_

7 Ag_2_O + I_2_ + O_2_ ↔ AgIO_3_ + AgI

8 Ag_2_O + I_2_ ↔ 2AgI + 0.5O_2_

**Fig. 4:**
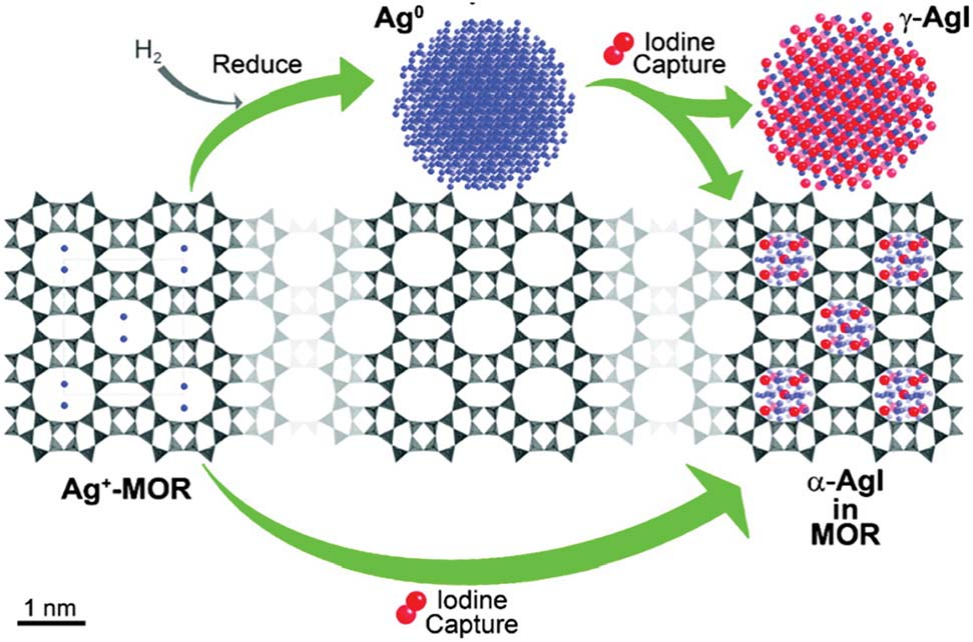
Schematic of capture of iodine by a silver-containing MOR-type zeolite.^171^ After a pre-reduction step using hydrogen, AgMOR gives rise to a mixture of γ-AgI nanoparticles and sub-nanometer α-AgI, whereas the direct uptake of iodine by silver-exchanged MOR exclusively produces sub-nanometer α-AgI. Reprinted with permission from K. W. Chapman, P. J. Chupas and T. M. Nenoff, *Journal of the American Chemical Society*, 2010, **132**, 8897–8899. Copyright 2010 American Chemical Society.

More recently, Chapman *et al.*^171^ showed the existence of several AgI species in the same AgMOR/I_2_ system. The structure adopted by silver iodide, as well as the distribution of different phases, depends on the temperature and the initial oxidation state of silver.^181^ At temperatures below 147 °C β-AgI (wurtzite structure) is the more stable phase, whereas at temperatures above 147 °C α-AgI (cubic centred structure) becomes the more stable form. However, at high temperatures a third phase can co-exist, namely, the metastable γ-AgI phase.^181^ The authors stated that silver is present in the form of Ag^0^ particles with sizes of 3 nm on the zeolite surface. Chapman *et al.*^171^ observed two AgI phases after the adsorption of I_2_: α-AgI, which was mainly localized in the pores of the silver zeolite, and the metastable γ-AgI phase, which was characterized by larger nanoparticles that were mostly localized on the zeolite surface (Fig. 4). According to the authors, these results involve the migration of silver in the zeolite pores during the adsorption of iodine. This migration is made possible by the mobility of both the iodine species (which are initially physisorbed into the pores) and the Ag^+^ ions, which are localized directly inside the zeolite framework. In the absence of pre-treatment with hydrogen, the non-reduced silver localized inside the mordenite gives rise to the formation of AgI confined in the zeolite pores in the form of nanoscale α-AgI clusters.

**Fig. 5:**
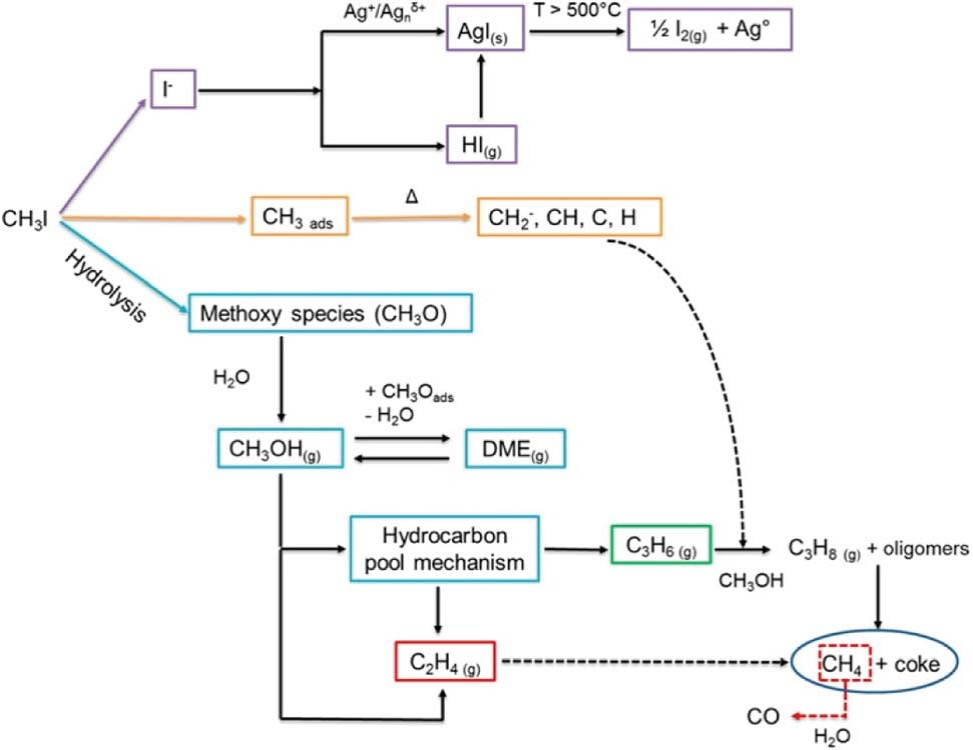
Different reaction pathways and mechanisms for the adsorption and decomposition of CH_3_I on AgY zeolite.^78^ Reprinted with permission from M. Chebbi, B. Azambre, L. Cantrel and A. Koch, *Journal of Physical Chemistry C*, 2016, **120**, 18694–18706. Copyright 2016 American Chemical Society.

In 2014, in a study that combined several *ex situ* characterization techniques, Nenoff *et al.*^173^ proposed a mechanism for the trapping of CH_3_I by silver-exchanged MOR-type zeolite (Ag^0^MOR, in which silver is reduced to its Ag^0^ form). This mechanism involves the acidic sites of mordenite and the catalytic decomposition of hydrocarbons derived from CH_3_I. In this study, the emission of various organic compounds was detected, such as dimethyl ether (DME), methanol, and methyl nitrite. These observations are consistent with a previous work by Heafner and Watson,^182^ in which the presence of methanol in the off-gas stream was reported. Scheele^29^ also suggested that methanol and DME are secondary products formed during the adsorption of CH_3_I onto silver. Therefore, Nenoff *et al.*^173^ explained that CH_3_I decomposed on the Brönsted acid sites (Si–OH–Al) of the zeolite to form adsorbed methoxy species (CH_3_O–), which react with water to form methanol and consequently free the acidic sites of the zeolite. Dimethyl ether was observed after the reaction of methanol with the adsorbed methoxy species. Finally, the iodine formed by the decomposition of CH_3_I is trapped in the form of an AgI precipitate. A recent work by Chebbi *et al.*^78^ confirmed the observations by Nenoff *et al.*^173^ (Fig. 5).

In addition, it was reported in several works that the formation of AgI in silver-exchanged zeolites was accompanied by the formation of various hydrocarbon compounds. Belapurkar *et al.*^31^ reported the formation of methane (CH_4_) and ethane (C_2_H_6_) during the adsorption of CH_3_I on AgX zeolites in dry conditions. The formation of alkanes (CH_4_ and C_3_H_8_) and alkenes (C_2_H_4_ and C_3_H_6_) is linked to the decomposition of chemisorbed CH_3_I species. In the literature, these species were observed during the decomposition of halogenated molecules (CH_3_I, CH_3_Cl) on supports such as AgX zeolites^31^ or aluminophosphates (HSAPO-34).^183^ The formation of hydrocarbons is a result of the direct coupling of fragmented hydrocarbon species, which is catalyzed by the active sites of the zeolite (silver sites and/or Brönsted acid sites). At higher temperatures, Chebbi *et al.*^78^ also observed the formation of carbon monoxide (*T* > 420 °C) and iodine species such as HI and I_2_ (*T* > 500 °C). The formation of carbon monoxide could originate from the partial oxidation of methane (or oxygenated species) by water, whereas the iodine species most probably originated from the reduction-oxidation decomposition of silver iodide (2AgI → 2Ag^0^ + I_2_).

Different silver phases (with different oxidation states) can be present in the pores of AgY zeolite, as shown by Chebbi *et al.*^78^ By using diffuse-reflectance UV-vis spectroscopy (DR-UV-vis), the authors obtained information on the nature of the silver species confined in the zeolite pores. Absorption peaks around 208–328 nm have been attributed to silver-exchanged Ag^+^ species, whereas absorption peaks beyond 305 nm are attributed to Ag^0^ nanoparticles. The distinction between Ag_*n*_^*δ*+^ and Ag_*m*_^0^ (*n* and *m* are the numbers of atoms in the clusters) is particularly difficult because the absorption spectra depend greatly on the sizes of the clusters. However, absorption peaks between 240 and 255 nm and at 305 nm are related to Ag_*n*_^*δ*+^ (*n* < 10) charged clusters and Ag_*m*_^0^ neutral clusters, respectively. To prove their assumptions and confirm the accurate localization of the silver species inside the zeolite framework, the authors^78^ studied the adsorption of CO at 35 °C by diffuse-reflectance infrared Fourier transform spectroscopy (DRIFT). This technique revealed that the silver species are well dispersed inside the framework of zeolites of the FAU structural type and that they are localized at the level of the exchanged active sites in the form of Ag^+^ cations. Metallic Ag_*m*_^0^ and charged Ag_*n*_^*δ*+^ aggregates (clusters) were also found near the exchanged active sites, but in lower quantities. During the exposure of the silver-exchanged zeolite to a CH_3_I flow at 100 °C, the Ag^+^ cationic species reacted with CH_3_I to form AgI precipitates. The formation of AgI involved the reaction of Ag^+^ with I^−^ after an initial step of the dissociation of CH_3_I. The dissociation of CH_3_I can occur on the silver active sites or the acidic sites of the zeolite. Then, AgI molecules tend to aggregate to form (AgI)_*n*_ nanoclusters. The sizes of the (AgI)_*n*_ aggregates increase continuously until they reach the size of the cavities and/or supercages of the zeolite. Chebbi *et al.*^78^ also studied the effect of water vapour and temperature on (AgI)_*n*_ clusters. They observed by different methods (XRD, DR-UV-vis) that some of these clusters migrated from the pores of the zeolite to its external surface. It was suggested that wet conditions promoted the formation of a microsolution inside the zeolite pores, which helped the silver to move in the pores and therefore permitted the coalescence or sintering of AgI species. To determine the reaction mechanisms, the desorption profiles of the different products and compounds formed during the decomposition of CH_3_I on the active sites of AgY zeolite (Brönsted acid sites, eqn (9) and (10), and/or directly on silver sites, eqn (11)) were also studied.

9 CH_3_I + H-zeolite → HI + CH_3_-zeolite

10 HI + Ag-zeolite → AgI + H-zeolite

11 CH_3_I + Ag-zeolite → AgI + CH_3_-zeolite

In the same context, Bučko *et al.*^172^ used computer simulations at the periodic DFT level to investigate the dissociative adsorption of iodomethane (CH_3_I) onto silver-exchanged mordenite (AgMOR). Using an *ab initio* molecular dynamics study, the authors investigated the structure, energetics and mobility of Ag(CH_3_I) complexes in the mordenite zeolite structure. In summary, the results indicated that the mechanism of the dissociation of CH_3_I proceeded more in accordance with eqn (11) than (9) + (10). AgI species formed during dissociative adsorption were shown to combine spontaneously into small (AgI)_*n*_ clusters, of which the dimensions were restricted by the size and geometry of the mordenite cages and pores.

To summarize, the formation of AgI precipitates is initiated by the dissociation of CH_3_I on the silver sites and/or Brönsted acid sites of the zeolite. AgI molecules and then (AgI)_*n*_ aggregates/clusters are formed inside the cages of zeolites of the FAU structural type. In the presence of humidity and at elevated temperatures, AgI precipitates can readily migrate and form larger entities on the external surface of the zeolite. Furthermore, the thermal and/or catalytic decomposition of CH_3_I (dissociation) leads to the production of various by-products of hydrocarbons such as methanol, DME, alkanes, and alkenes.

#### 2.2.2. Capture performance of Ag-exchanged zeolites

Numerous studies were carried out from the 1960s to the 1980s on the use of zeolites for the capture of radioactive iodine compounds in the event of nuclear accidents. In 1968 and 1970, Maeck *et al.* worked on more than twenty silver-impregnated zeolites in various operating conditions for the capture of iodine compounds (mainly I_2_ and CH_3_I).^38,40^ The best adsorption performance was achieved with AgX zeolite (FAU type). In comparison with silver-exchanged zeolites, additional studies confirmed that zeolites in which cations were exchanged for other metals such as Cu, Pd, and Cd have lower adsorption capacities and poorer performance (this phenomenon will be discussed below). Later, numerous studies were carried out on this topic, especially during the 1970s and 1980s^21,29,34,38,40,47,52,180^ and then between 2000 and 2017.^5,8,71,74–80,169–171,173–177,184–188^ These studies focused on the determination of the influence of various parameters such as the temperature (°C), silver content (wt%), Si/Al ratio, superficial velocity, and pretreatment of the material on the adsorption capacities of silver-exchanged zeolites (ESI, Table 3†). Other works focused on issues such as aging of the material under different atmospheres (NO_*x*_, humidity).^178,185^ Since 2016, we have observed a resurgence in studies on silver-exchanged zeolites for iodine capture. We can in particular mention the works by Chibani, Chebbi and Azambre,^74–80^ who performed systematic studies on the influence of zeolite parameters on their iodine adsorption capacities. These works, as well as those on the influence of the other parameters mentioned above, will be discussed in the following section.

#### 2.2.3. Influence of different parameters on the capture performance of Ag-exchanged zeolites

##### Influence of silver content

The silver content in an exchanged zeolite has a major influence on its adsorption capacity for iodine species. In 2001, Choi *et al.*^71^ studied the influence of the silver content in a faujasite-type zeolite (AgX) on its iodine adsorption capacity. They incorporated 10, 20 and 30 wt% silver by exchanging Na^+^ in NaX zeolite for Ag^+^. An increase in the adsorption capacity for iodine was observed when the silver content increased. At 100 °C, the adsorption capacity rose from 180 g kg_adsorbent_^−1^ to 250 g kg_adsorbent_^−1^ for a silver content of 10 wt% and 30 wt%, respectively. However, a large silver content (>20 wt%) entails a decrease in the pore volume, which in consequence limits the accessibility of the pores for iodine species (diffusion limitation).^71^ In fact, the micropore volume decreases from 2.37 × 10^−4^ m^3^ kg^−1^ for a non-exchanged zeolite to 1.83 × 10^−4^ m^3^ kg^−1^ in the case of the zeolite with 30 wt% Ag. The use efficiency of Ag was also estimated by calculating the Ag/I ratio. This ratio was optimal (>99%) for the zeolite with 10 wt% Ag, whereas it was only 85% and 68% for the zeolites with 20 wt% and 30 wt% Ag, respectively, at temperatures between 150 and 200 °C. Because silver is expensive, there is interest in the incorporation of an optimal silver content with the highest efficiency. More recently, Cheng *et al.*^188^ studied the adsorption of I_2_ on a silver-exchanged FAU-type zeolite (AgX) at temperatures between 250 and 650 °C. They observed a decontamination factor (DF) in the range of >10^3^ for a silver content of greater than 15 wt%, in contrast to 10^1.5^ to 10^3^ for a silver content of less than 15 wt%. However, only slight differences in the decontamination factor were observed between exchanged zeolites with 15 wt% Ag and 20 wt% Ag, which seems to be in agreement with the study by Choi *et al.*,^71^ which implies that there was no real increase in the adsorption capacity at a high silver content. In studies by Chebbi *et al.*^77^ and Azambre *et al.*^74^ on several silver-exchanged zeolites (AgY, AgX, AgMOR, AgZSM-5, *etc.*) with different silver contents (0 to 35 wt%) at 100 °C, an increase in the adsorption capacity of silver-exchanged FAU-type zeolites (AgY and AgX) from 87 mg g_adsorbent_^−1^ to 223 mg g_adsorbent_^−1^ for a silver content of 9.1 wt% to 22.8 wt% in AgY and from 149 mg g_adsorbent_^−1^ to 267 mg g_adsorbent_^−1^ for a silver content of 7.3 wt% to 35 wt% in AgX was observed.

##### Influence of the zeolite type

**Fig. 6:**
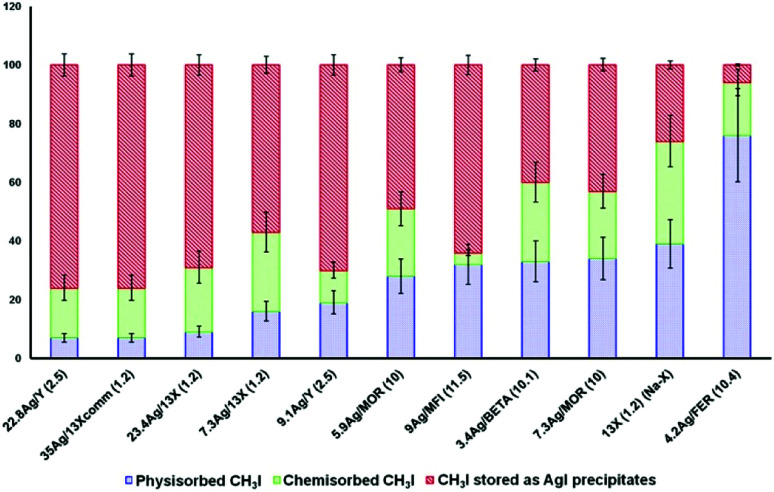
Ranking of the ability of different sorbents to promote the irreversible trapping of CH_3_I as AgI. The best sorbents are on the left side of the diagram. Reprinted with permission from B. Azambre and M. Chebbi, *ACS Applied Materials and Interfaces*, 2017, **9**, 25194–25203. Copyright 2017 American Chemical Society.

Studies for a long time mainly focused on zeolites of the FAU and MOR structural types.^5,8,29,31,34,38,40,47,52,71,169–171,173,175,177,178,182,184,185,187,188^ The interest in these two types of zeolites can be explained by their large pore openings, which allow easier diffusion of iodine (I_2_, CH_3_I) and non-iodine (NO_*x*_, CO_*x*_, H_2_O, *etc.*) compounds inside the zeolites. Other zeolites, such as the MFI, *BEA and FER structural types, could potentially be suitable for iodine adsorption applications. After the study by Chebbi *et al.*^77^ on the influence of the silver loading, it was concluded that AgX zeolite exhibited the best performance in the capture of iodine compounds, followed closely by AgY, whereas the performance of MFI, *BEA and FER zeolites was not of as much interest. For a similar silver content (∼9 wt%), AgZSM-5 and AgY have similar adsorption capacities (∼85–87 mg g_adsorbent_^−1^). However, these results differ greatly from those observed for AgX zeolite, which exhibited a saturation capacity of 149 mg g_adsorbent_^−1^ for a silver content of 7.3 wt%. The discrepancy between these silver-exchanged zeolites indicated that the silver content is not the only parameter that has to be considered for the adsorption of iodine species. Accordingly, AgX and AgY zeolites exhibit the best performance, which is principally due to their higher silver contents, in contrast to the other zeolites with low silver contents studied by Chebbi.^77^ Moreover, Chebbi *et al.*^77^ highlighted the fact that AgX zeolite has a cation exchange capacity that is much higher than that of AgY zeolite (iodine adsorption capacities of 234 mg g_adsorbent_^−1^ for a silver content of 23.4 wt% and 267 mg g_adsorbent_^−1^ for a silver content of 35 wt% in AgY and AgX zeolite, respectively), thanks to its low Si/Al ratio. In conclusion, AgX zeolite was estimated to be the best silver-exchanged zeolite in terms of performance. However, Azambre *et al.*^74^ and Chebbi *et al.*^77^ carried out further studies by examining the quantities of physisorbed and chemisorbed iodine species and those captured in the form of AgI precipitates (AgI) to determine which silver-exchanged zeolites display the best performance in the irreversible capture of iodine. By temperature-programmed desorption experiments in combination with FTIR spectrometry, they studied the adsorption and desorption profiles of CH_3_I on different silver-exchanged zeolites (AgX, AgY, AgZSM-5, AgMOR, AgFER and AgBEA) to determine the quantities of physisorbed and chemisorbed iodine (CH_3_I) at saturation (Fig. 6).

The reversibility of the capture of CH_3_I differs drastically depending on the structural type of the silver-exchanged zeolite considered.^77^ X and Y faujasite zeolites that have large pore openings with a size of 7.4 × 7.4 Å and a silver loading of greater than 20 wt% exhibit the best performance in terms of iodine capture, and more than 90% of CH_3_I is irreversibly adsorbed (chemisorbed + AgI precipitate). In contrast to faujasite-type zeolites, the proportion of physisorbed CH_3_I on impregnated AgFER zeolite (4.2 wt% Ag) with pore sizes of 3.5 × 4.8 Å and 4.2 × 5.4 Å is significantly higher (76 ± 16%). This behaviour cannot only be attributed to the low loading of silver, given that in the case of Ag*BEA zeolite (3.4 wt% Ag) with pore sizes of 5.6 × 5.6 Å and 6.6 × 6.7 Å the physisorbed component comprises only 33 ± 7%. The kinetic diameter of CH_3_I (between 5 and 6 Å) is far greater than the pore sizes of AgFER zeolite (3.5 × 4.8 Å and 4.2 × 5.4 Å), which could partly explain why physisorption is favoured at the expense of the formation of an AgI precipitate. The structure of the zeolite seems to have a non-negligible influence on the adsorption capacity and retention of iodine species, as zeolites with small pores favour physisorption, whereas zeolites with larger pores favour retention by chemisorption and the formation of an AgI precipitate.^77^

##### Influence of the Si/Al ratio in the zeolite framework

**Fig. 7:**
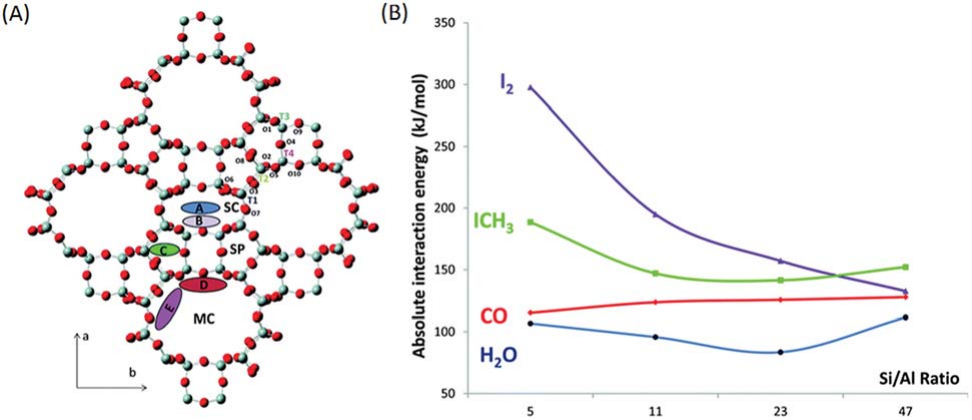
(A) Schematic of the different channels present in the mordenite structure: main channel (MC), side pocket (SP) and side channel (SC). The potential locations of aluminium atoms at the four non-equivalent tetrahedral sites T1 to T4 are indicated. The capital letters A–E correspond to different Mortier sites, which can be occupied by extra-framework Ag^+^ cations. Colour code: O atoms are in red and Si atoms are in pale blue. (B) Average interaction energies of CH_3_I, I_2_, CO and H_2_O at T1-E sites as a function of the Si/Al ratio. Reproduced from ref. 79 with the permission of the PCCP Owner Societies.

The amounts of silver that can be exchanged in a zeolite greatly depend on the Si/Al ratio in the zeolite framework. In fact, Chebbi *et al.*^77^ and Azambre *et al.*^74^ obtained silver loadings of greater than 20 wt% after several cation exchange steps in NaX- and NaY-type zeolites. However, in the case of MFI-, MOR-, FER- and *BEA-type zeolites the silver loading never exceeded 10 wt%. The discrepancies between these structures can be linked to the fact that FAU-type zeolites have a low Si/Al molar ratio in the framework (1 < Si/Al < 3), which leads to a higher cation exchange capacities. Moreover, a study performed using density functional theory (DFT)^79^ has shown that the interaction energies of iodine compounds increase considerably when the Si/Al ratio decreases (Fig. 7). The interaction energy increases from 145 kJ mol^−1^ to 190 kJ mol^−1^ and from 135 kJ mol^−1^ to 300 kJ mol^−1^ for CH_3_I and I_2_, respectively, as the Si/Al ratio decreases from 47 to 5. Chibani *et al.* proved *via* this study that (1) for a zeolite framework with a low Si/Al ratio contaminants such as H_2_O and CO have little or no effect on the adsorption capacities of silver for iodine compounds and (2) at the same Si/Al ratios iodine compounds interact more strongly with silver, which enables the dissociation of I_2_ and formation of AgI for the immobilization of iodine. Therefore, the Si/Al ratio has a strong influence on the iodine adsorption capacity. Hence, a low Si/Al ratio has two advantages: it provides greater silver exchange capacities and favours interactions between silver and iodine compounds.

##### Influence of pretreatment with hydrogen

Several studies have established that the pretreatment of silver-exchanged zeolites with hydrogen significantly improves their performance in the capture of CH_3_I and I_2_. In 1978, Thomas *et al.*^60^ reported an increase in the adsorption capacity for I_2_ by a factor of two after pretreatment with hydrogen (5 L min^−1^ H_2_, 500 °C, 24 h) for silver-exchanged mordenite (Ag^0^MOR, *Q* = 138 mg_I_2__ g_Ag_^−1^, bed length 2.5 cm) in comparison with a non-pretreated silver-exchanged mordenite (AgMOR, *Q* = 71 mg_I_2__ g_Ag_^−1^, bed length 2.5 cm). Subsequently, these results were confirmed by Jubin^28,64^ and Scheele *et al.*^29^ for the same type of zeolite. In 1980, studies led by Jubin^28^ showed that pretreatment with H_2_ (100% H_2_) at high temperatures (200, 400 and 500 °C) for a long duration (24 h and 48 h) led to a decrease in the adsorption capacity for iodine compounds (CH_3_I). At 200 °C, the adsorption capacity decreased from 35.6 mg_CH_3_I_ g^−1^ to 12.3 mg_CH_3_I_ g^−1^ for a pretreatment duration of 24 and 48 hours, respectively. The same results were obtained at higher temperatures (400 °C and 500 °C). This sudden decrease in performance is explained by the formation of silver nanoclusters and large aggregates, which limits the accessibility of silver. Therefore, it is necessary to define accurately the two parameters of pretreatment (temperature and duration) to maximize the adsorption capacities of silver-exchanged zeolites. With this in mind, Nan *et al.*^174^ recently determined the optimal temperature for the pretreatment with H_2_ of silver-supported mordenite (AgMOR, 12 wt%) for the adsorption of iodine (I_2_). They showed that after reduction by hydrogen (4% H_2_ in argon, 500 mL min^−1^) for 24 hours the iodine adsorption capacity of Ag^0^MOR increased when the pretreatment temperature was increased from 170 °C to 400 °C. However, after the temperature reached 400 °C the adsorption capacity did not increase further. Studies by Zhao *et al.*^177^ and Aspromonte *et al.*^169^ confirmed these results.

##### Influence of the operating temperature

Temperature is a crucial parameter that can significantly influence the adsorption capacities for iodine compounds of a silver-exchanged zeolite. In 1982, Vance *et al.*^34^ studied the thermal stability in iodine capture of the silver-exchanged zeolites AgY (28 wt%), AgX (37 wt%) and AgMOR (20 wt%). These three zeolites were first saturated with iodine under dry conditions at 130 °C. Then, the temperature was increased from 130 °C to 1300 °C to observe the effect of temperature on the iodine content in these zeolites. A significant decrease in the iodine content captured in the zeolites was observed upon an increase in the temperature. The iodine content in AgY zeolite (28 wt%) decreased monotonically from 210 mg_I_2__ g_adsorbent_^−1^ to 50 mg_I_2__ g_adsorbent_^−1^ at temperatures of 130 °C and 1300 °C, respectively. However, it can be observed that at temperatures above 700 °C the zeolite framework generally tends to collapse and consequently loses crystallinity and becomes amorphous. This could explain the significant decrease in the iodine content observed by these authors.^34^ In 2001, the influence of temperature on AgX zeolite loaded with 10, 20 and 30 wt% silver was studied by Choi *et al.*^71^ An increase in temperature led to a decrease in the adsorption capacities of the zeolite for iodine compounds. For AgX zeolite (10 wt%), the adsorption capacity decreased from 180 mg g_adsorbent_^−1^ to 130 mg g_adsorbent_^−1^ at temperatures of 100 °C and 400 °C, respectively. Similar conclusions were reached by Belapurkar *et al.*^31^ regarding the adsorption capacities for CH_3_I of dehydrated AgX zeolites at temperatures between 25 and 150 °C. Furthermore, Cheng *et al.* observed a relatively significant decrease in the decontamination factor (DF) for silver-exchanged AgX zeolite between 250 °C and 650 °C. In the case of a 15 wt% AgX zeolite, the decontamination factor decreased from 10^3.4^ to 10^3.0^ as the temperature was increased from 250 °C to 650 °C.

From all these studies, it should be expected that the iodine adsorption capacity of a silver-exchanged zeolite decreases with temperature. However, Nan *et al.*^174^ observed an increase in the adsorption capacity of silver-reduced Ag^0^MOR (12 wt%) upon an increase in the temperature from 100 °C to 150 °C. Nevertheless, the adsorption capacity still decreased at temperatures above 150 °C. Nan *et al.* linked this behaviour to the influence of water on the adsorption capacity and properties of mordenite. In fact, various studies have reported that water has a negative effect on the adsorption capacity for iodine. It was reported that the influence of water is less pronounced at higher temperatures than at lower temperatures. The probability that silver will react with water to form silver oxide (Ag_2_O) or its hydroxide decreases. Furthermore, iodine compounds have easier access to the pores and adsorption sites of the zeolite.

##### Influence of superficial velocity

Another factor that affects iodine adsorption performance is the flow rate of the carrier gas through the zeolite bed. According to Scheele *et al.*,^29^ the flow rate is governed by four parameters, namely, the superficial velocity, bed diameter, bed length and residence time. Few studies have examined the effects of these parameters on the iodine capture performance of silver-exchanged zeolites. The bed diameter and length and the superficial face velocity and contact time are interrelated. In a study by Scheele *et al.*,^29^ the authors decided to ignore potential effects of the bed size and contact time by arbitrarily selecting a bed diameter and bed length to exceed the expected zone of mass transfer and thus regrouped these factors into one variable, namely, superficial face velocity. In their work, the authors^29^ confirmed that an increase in the superficial velocity from 3.75 m min^−1^ to 15 m min^−1^ decreased the adsorption capacities of AgMOR zeolites for CH_3_I from 71 mg g_adsorbent_^−1^ to 7 mg g_adsorbent_^−1^, respectively. This trend was also observed by Pence *et al.*^40^ for AgX-type zeolites. A thorough knowledge of the related operating conditions in reactors and the associated kinetics is crucial for maximising the adsorption capacities of silver-exchanged zeolites.

##### Influence of inhibitors

During a severe nuclear accident, several compounds such as H_2_O, CO, CO_2_, nitrogen oxides (NO_*x*_), hydrocarbons and halogenated organic compounds are released. These compounds can act as inhibitors and poisons and thus limit the iodine adsorption capacities of silver-exchanged zeolites.

###### Influence of water

First of all, the influence of water and humidity on a silver-exchanged zeolite was studied. In 1976, Thomas *et al.*^52,60^ showed that very little variation in the iodine (I_2_) adsorption capacity of Ag^0^MOR was observed in the presence of water (dew point 35 °C) in the carrier gas flow (4 ± 12 mg_I_2__ g_AgMOR_^−1^). They concluded that the presence of water did not affect or only slightly affected the I_2_ trapping performance, which was contradicted by more recent studies. The effect of water on the adsorption capacity for CH_3_I was also studied by Scheele and Burger.^29,32^ The authors demonstrated that a water concentration of around 5 × 10^−4^ mol L^−1^ in the carrier gas was beneficial for the adsorption capacity for CH_3_I (*Q* = 139 g kg_adsorbent_^−1^) rather than a lower concentration of 4.3 × 10^−6^ mol L^−1^ (*Q* = 30 g kg_adsorbent_^−1^). These observations were confirmed by Jubin,^28,64^ who asserted that the presence of a moderate amount of water increased the adsorption capacities for CH_3_I of Ag^0^MOR zeolites, whereas a dry atmosphere or high relative humidity led to a decrease in the adsorption capacities. Water in the carrier gas seems to be beneficial for increasing the adsorption capacities of silver-exchanged zeolites. However, no advanced studies have determined the exact conditions of humidity for the optimal utilization of silver-exchanged zeolites.

The adsorption capacities for CH_3_I were also studied by Belapurkar *et al.*^31^ for temperatures between 25 and 150 °C and degrees of hydration between 0 and 18 wt%. They determined that humidity has a negative effect on performance. This was particularly significant at low temperatures, as a decrease in capacity of 56% was observed at 25 °C in contrast to 17% at 150 °C. Further studies by Belapurkar *et al.*^31^ showed that in the absence of silver (NaX) the effect of hydration on adsorption capacities is far more pronounced than in the case of a silver-exchanged zeolite (AgX). This discrepancy between the two zeolites can be explained by the fact that in zeolites in which no cations are exchanged for silver (NaX) adsorption phenomena are dominated by physisorption. In fact, notably at low temperatures, competition between water molecules and CH_3_I species occurs for adsorption sites. Consequently, the adsorption capacities for CH_3_I are reduced. In the case of silver-exchanged zeolites, chemisorption phenomena are prevalent. Therefore, the effect of water is reduced, especially at high temperatures.

In a similar way, Choi *et al.*^185^ demonstrated that water (relative humidity of 50%) had a negative effect on the decontamination factor of an AgX zeolite (DF = 3 × 10^4^ to DF = 2 × 10^3^ at 200 °C). Finally, Jubin *et al.*^189^ studied the effect of long-term aging (up to 6 months) of a commercial Ag^0^MOR zeolite under dry and humid conditions before the adsorption of I_2_. After exposure to a dry atmosphere for 6 months, a decrease of 40% in the iodine adsorption capacity was observed. On the other hand, in the case of exposure to a humid atmosphere a decrease of 45% in the iodine adsorption capacity was observed in only 1 month. The authors explained that aging in dry conditions for 6 months is equivalent to aging for 1 month in humid conditions, which underlined the strong effect of water on the adsorption capacities of silver-exchanged zeolites. In conclusion, despite a certain amount of contradiction in the literature, the general effect of water and/or humidity on the adsorption capacities of silver-exchanged zeolites seems to be negative. However, a low concentration of water is believed to favour the mechanism of the trapping of CH_3_I on silver.

###### Influence of NO_*x*_

Nitrogen oxides, which are more commonly known as NO_*x*_, are acknowledged by the scientific community to influence the retention and capture performance of silver-exchanged zeolites.^28,63,64^ NO_*x*_ have a more or less adverse effect depending on the iodine species in consideration, their concentrations, the type of zeolite used and the operating conditions (superficial velocity, temperature, presence of other contaminants). Even though several studies reported ambiguous or conflicting results, the majority agreed on the fact that NO_*x*_ impair to a certain extent the adsorption performance of silver-exchanged zeolites. In 1979, Holladay^63^ studied the effect of NO and NO_2_ on the iodine retention capacity of silver-exchanged mordenite (Ag^0^MOR). In the absence of NO_*x*_ contaminants, the capacity for iodine that was measured was 113 ± 12 mg g_Ag^0^MOR_^−1^. Surprisingly, when 2% NO was added to the carrier gas the adsorption capacity of silver-exchanged mordenite increased to 129 ± 10 mg g_Ag^0^MOR_^−1^. However, when 2% NO_2_ was added to the feed gas, this time a decrease in the adsorption capacity could be observed (68 ± 12 mg g_Ag^0^MOR_^−1^). The author^63^ assumed that NO acts as a reducing agent in the presence of oxygen to maintain silver in its highly reactive reduced metallic form (Ag^0^). However, the presence of NO_2_ allows the slow oxidation of silver to silver oxide (Ag_2_O), which is less reactive and would consequently decrease the adsorption capacity. The presence of both compounds (NO + NO_2_) in the carrier gas slightly modified the adsorption capacity (119 ± 12 mg_I_2__ g_Ag^0^MOR_^−1^). Jubin^28,64^ also carried out experiments on the adsorption of CH_3_I onto an Ag^0^MOR zeolite in the presence of NO_*x*_ (0–3% NO and 0–3% NO_2_). The results indicated that the presence of NO_*x*_ slightly modified the adsorption performance. Recent studies led by Bruffey *et al.*^178^ finally helped to conclude that the presence of nitrogen oxides such as NO_*x*_ ([NO_*x*_] = 10 000 ppm in air) has a negative effect on the adsorption capacities of silver-exchanged zeolites for CH_3_I, with a decrease from 125 mg_CH_3_I_ g_Ag^0^MOR_^−1^ to 56 mg_CH_3_I_ g_Ag^0^MOR_^−1^. In the presence of NO and NO_2_ in the carrier gas, the adsorption capacity for CH_3_I decreases by 40% in comparison with the adsorption capacity determined in dry air alone. Furthermore, the presence of NO_*x*_ allows the oxidation of CH_3_I to I_2_ (ref. 178) according to the following thermodynamically favoured reactions^29^ (eqn (12), Δ*G* = −1079 kJ mol^−1^, *T* = 400 K and eqn (13), Δ*G* = −853 kJ mol^−1^, *T* = 400 K):

12 7NO + 2CH_3_I ↔ I_2_ + 3H_2_O + 2CO_2_ + 7/2N_2_

13 7NO_2_ + 4CH_3_I ↔ 2I_2_ + 6H_2_O + 4CO_2_ + 7/2N_2_

**Fig. 8:**
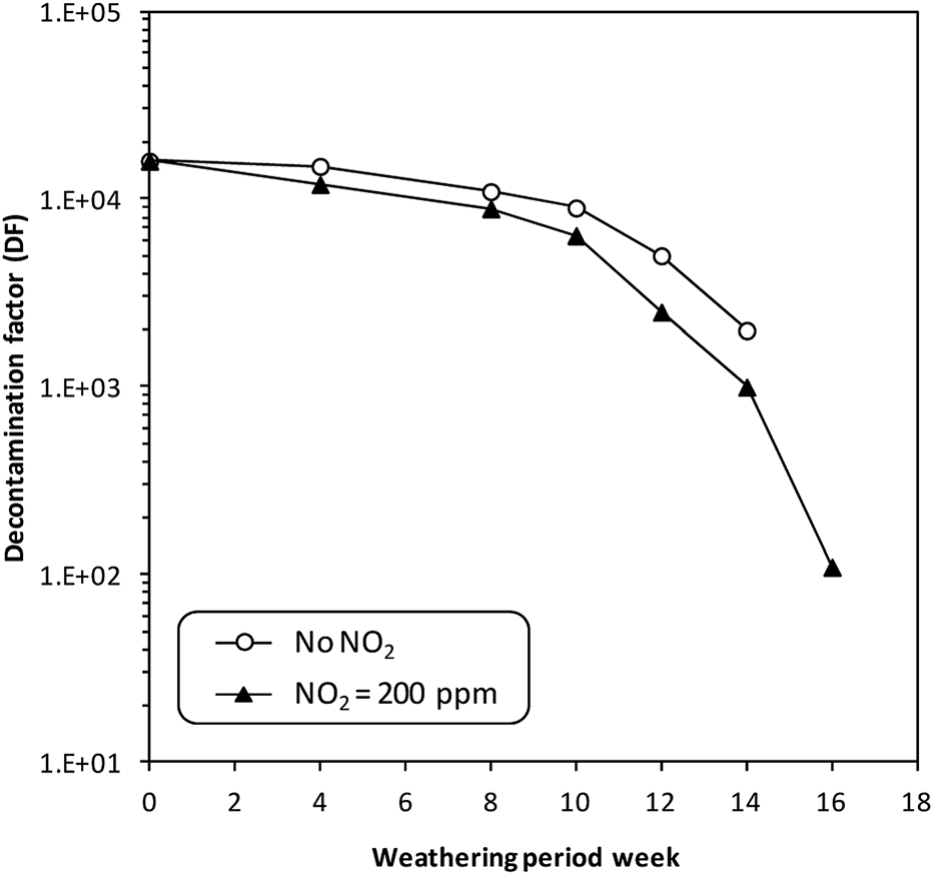
Effect of long-term weathering on the decontamination factor for the retention of radioactive methyl iodide using a 10 wt% AgX adsorbent with NO_2_ gas poisoning. Adapted by permission from Springer Nature from B. S. Choi, G. Il Park, J. W. Lee, H. Y. Yang and S. K. Ryu, *Journal of Radioanalytical and Nuclear Chemistry*, 2003, **256**, 19–26, Copyright 2003.

However, no experimental studies have proved the occurrence of such reactions during tests on the adsorption of CH_3_I and I_2_ in the presence of NO_*x*_. Studies of the long-term influence of NO_2_ (200 ppm) on the adsorption capacity of a silver-exchanged X zeolite (10 wt% Ag) were carried out by Choi *et al.*^185^ These studies demonstrated that no significant effect could be observed on the adsorption performance of the zeolite in the presence of NO_*x*_ in the short term (several weeks). However, in the long term, *i.e.*, after poisoning by NO_*x*_ for 16 months, the adsorption efficiency declined from 99.9% (DF = 10^4^) to 99.0% (DF = 10^2^) (Fig. 8).

Bruffey *et al.*^175^ studied the iodine trapping stability of an Ag^0^MOR zeolite in the presence of 2% NO_2_ for a duration of 1 to 4 months. The authors initially saturated the zeolite with iodine to give an average adsorption capacity of 72 mg_I_2__ g_Ag^0^MOR_^−1^. Then, aging and stability tests were carried out at 150 °C with 2% NO_2_ in the carrier gas for 1, 2, 3 and 4 months. The different tests showed an absence of released iodine after exposure to NO_*x*_ for 4 months. In conclusion, the presence of nitrogen oxide (NO_*x*_) in the carrier gas or the surrounding atmosphere is believed to affect negatively the adsorption performance of silver-exchanged zeolites, but the effect is not drastic. According to the literature, this could be attributed to a modification of the oxidation state of silver (Ag^0^ to Ag_2_O). However, in the case of a few studies the presence of NO_*x*_ did not have any important effect on the iodine adsorption capacities of zeolites. Furthermore, aging studies show a more pronounced adverse effect in the long term rather than in the short term.

###### Influence of organics and halogen compounds

Organics and halogen compounds are also acknowledged to have an influence on the adsorption capacities of silver-exchanged zeolites. Jolley *et al.*^30^ studied the effect of various volatile organic compounds (alkanes, alkenes, aromatics, ketones, alcohols, and chlorine- and bromine-containing compounds) on the efficiency of the adsorption of CH_3_I by silver-exchanged X zeolite (AgX). The adsorption capacity for CH_3_I was unaffected or only slightly affected by small molecules (such as hexane or ethanol), whereas in the case of molecules with larger sizes the performance of the silver-containing material was greatly affected in a negative way. Different families of organic compounds had the following inhibitory effects on the adsorption capacity of AgX zeolite: alkynes > alcohols > ketones and aromatics > alkanes; alkynes had the most detrimental influence on the adsorption capacity. The size of the molecule appears to play an important role, as it sterically hinders access to the zeolite pores. Therefore, the capture of iodine by silver inside the zeolite pores is limited (diffusion limitation). However, this steric hindrance effect is not the only factor in the decrease in the adsorption capacity of the silver-exchanged zeolite. In fact, heteroatoms such as chlorine, bromine and oxygen and/or unsaturated compounds can be adsorbed onto silver sites thanks to the strong interactions that exist between them and silver. Halogenated compounds, especially chlorine-containing compounds, tend to dissociate and release chlorine, which will poison the silver active sites. Surface analysis techniques (XPS, EDX) have shown that the adsorption of halogenated compounds (such as chloromethane or bromomethane) gives rise to the formation of metallic silver halide precipitates (AgCl and AgBr). From a thermodynamic point of view, chlorine is the main inhibitor of the adsorption of iodine onto silver-exchanged zeolites. Undeniably, because an AgCl precipitate (Δ_f_*G*° = −109.9 kJ mol^−1^) is more stable than an AgI precipitate (Δ_f_*G*° = −66.3 kJ mol^−1^), the presence of a large excess of Cl_2_, HCl or CH_3_Cl in the environment near a silver-exchanged zeolite significantly affects and impairs its performance. Burchsted *et al.*^56^ reported in 1976 that a silver-exchanged zeolite bed became totally ineffective for the capture of iodine nearly instantly after HCl was introduced into it. Similar studies by Ackley *et al.*^49^ showed that the presence of chlorine (Cl_2_) significantly impaired the iodine adsorption performance of an AgX zeolite in terms of the decontamination factor (DF). They reported for a silver-exchanged zeolite that was exposed to Cl_2_ concentrations between 0.4 and 0.8 mmol mL^−1^ that the decontamination factor for a bed length of 10 cm decreased to DF = 14–20 in contrast to DF = 10^2^ in the absence of chlorine. In conclusion, organic compounds, especially halogenated compounds, have a strong influence on the iodine adsorption capacities of silver-exchanged zeolites. It can be observed that chlorine is the most strongly inhibiting compound for this type of material, which is principally due to its high affinity for silver as shown by the formation of a stable AgCl precipitate.

###### Influence of other compounds

Other compounds can also be present during a severe nuclear accident, such as CO_*x*_ (CO_2_ and CO), SO_*x*_ (SO_2_ and SO_3_) and, to a lesser extent, P_4_O_10_.^186^ These compounds, which have not been studied in depth, seem to not significantly affect the iodine adsorption performance of silver-exchanged zeolites. However, in their presence, the diffusion of iodine species inside zeolite pores might become limited for steric reasons.

Despite a few contradictions in the literature, most studies acknowledged that the inhibitors mentioned previously (H_2_O, NO_*x*_, organics, halogenated compounds, CO_*x*_, SO_*x*_, *etc.*) generally have a negative effect on the iodine adsorption capacities of silver-exchanged zeolites. A recent study performed by Chebbi *et al.*^81^ confirmed all the observations cited above.

##### Influence of γ-radiation

Little information can be found in the literature on the influence of γ-radiation on the iodine adsorption performance of silver-exchanged zeolites.^59^ Evans^59^ studied an AgX zeolite, which had been saturated with iodine beforehand, in the presence of γ-radiation for exposure durations of up to 104 hours. These tests demonstrated that at temperatures of >45 °C and in a water-rich atmosphere the destabilisation of trapped I_2_ is favoured. The desorption rate was 0.1% per hour at a temperature of 80 °C and a relative humidity of 90%. After desorption for 105 hours (in various operating conditions: 35 °C ≤ *T* ≤ 80 °C and 20% ≤ R.H. ≤ 95%), the total quantity of iodine released remained fairly low (approximately 0.74% of the initial quantity of iodine trapped beforehand). These results show that the stability of iodine trapped in silver-exchanged zeolites (AgX) is not significantly affected by the presence of γ-radiation. Recent studies by Chebbi *et al.* confirmed the absence of an influence of γ-radiation on the iodine adsorption capacities of AgY and AgX zeolites.^81^

All these studies that were performed on silver-exchanged zeolites used for iodine capture and carried out under different operating conditions (inlet iodine concentration, bed length, *etc.*) are difficult to compare. Furthermore, in some studies the zeolites used were not completely crystalline. In addition, sometimes commercial and home-made zeolites with different loadings of silver were compared, which is not appropriate.

#### 2.2.4. Zeolites loaded with other metals for the capture of radioactive iodine

One of the main drawbacks of silver-exchanged zeolites is the inherent cost of the silver metal used. For this reason, other elements have been studied to obtain low-cost metal-exchanged zeolites for the adsorption of iodine. Between 1968 and 1970 Maeck and Pence^38–40^ studied the iodine capture performance of approximately twenty zeolites impregnated with metals (Ag, Na, Cu, Pd, Tl, *etc.*). Their studies on NaX zeolite proved the great superiority of silver in terms of iodine adsorption over the other elements. The silver-exchanged zeolite (AgX) exhibited an iodine adsorption efficiency of 99.9%, whereas those of the other exchanged zeolites were less than 30%. No elements other than silver proved to have acceptable performance for the adsorption of iodine. After these works, Staples *et al.*^52^ studied the effect of the exchanged cations (Na^+^, Ag^+^, Pb^2+^, Cd^2+^) on the irreversibility of the capture of iodine (I_2_) by the faujasite-type X zeolite at 150 °C. Even though the iodine adsorption capacity of every cation was acceptable, only silver was capable of trapping iodine in an irreversible way. Recent studies,^75,76,79,80^ especially those using density functional theory (DFT) simulations, enabled the determination of the influence of the valence of cations on the selective adsorption of iodine species by zeolites. In an initial study, Chebbi *et al.*^75^ showed that copper (Cu^+^)- and silver (Ag^+^)-exchanged faujasite zeolites (CuX and AgX, respectively) preferentially adsorbed iodine compounds rather than species such as water, chloromethane and chlorine. Furthermore, the adsorption of iodine species is much more pronounced on the CuX and AgX zeolites rather than on the protonated or sodium forms of faujasite zeolites. However, the presence of inhibitors such as carbon monoxide (CO) or nitrogen oxide (NO) limits the selectivity of Cu^+^ zeolites for the adsorption of iodine. Despite its good performance in the adsorption of iodine, CuX zeolite cannot be used in the presence of elevated quantities of CO or NO, which would be present during a severe nuclear accident. In contrast to CuX zeolite, AgX zeolite displayed good adsorption of iodine species in the presence of NO. It can be observed that these modelling results do not agree totally with the experimental results described in the section on the influence of inhibitors. In a second study, the influence of the presence of Cu^2+^, Pb^2+^, and Hg^2+^ in mordenite zeolite on its iodine adsorption performance, with a comparison of the adsorption energies of CH_3_I and I_2_, was investigated. In the presence of large quantities of contaminants (H_2_O, CO, CH_3_Cl, Cl_2_), HgMOR was found to be the best adapted metal-exchanged mordenite for the adsorption of iodine. From a thermodynamic point of view, the tendency of a metal to form the corresponding halide (MI) can be estimated from the Gibbs free energy of formation (Δ_f_*G*°, kJ mol^−1^). By comparing the energies of formation of halides and the corresponding oxides, it is possible to identify the most thermodynamically stable compounds. Table 1 lists the Gibbs free energies of formation of several halides and oxides.^190,191^

**Table 1:** Comparison of Gibbs free energies of formation of iodides and oxides (Δ_f_*G*°, kJ mol^−1^)^190,191^

Metal	Iodide	Δ_f_*G*° (kJ mol^−1^, 25 °C)	Oxide	Δ_f_*G*° (kJ mol^−1^, 25 °C)
Ag	AgI	−66.3	Ag_2_O	−11.2
Cu	CuI	−69.4	CuO	−129.8
Hg	HgI_2_	−102.3	HgO	−58.6
	Hg_2_I_2_	−111.2	HgO	−58.6
Fe	FeI_2_	−109.1	FeO	−246.5
Tl	TlI	−125.5	Tl_2_O	−147.3
Sn	SnI_2_	−144.3	SnO	−257.0
	SnI_4_	−215.2	SnO_2_	−519.9
Cd	CdI_2_	−201.4	CdO	−228.6
Pb	PbI_2_	−173.6	PbO	−188.0
Ti	TiI_2_	−258.7	TiO_2_	−884.9
	TiI_4_	−370.9	TiO_2_	−884.9

In the majority of cases, the oxide will be preferentially formed rather than the halide form, except for silver (Ag) and mercury (Hg). It is therefore not surprising that in most studies the authors observed that other metals had low adsorption capacities in comparison with that of silver. In conclusion, studies on zeolites in which cations were exchanged for various metals (Na, Cu, Pd, Cd, Tl, *etc.*) proved that their efficiency in the capture of iodine species is low and showed that silver-exchanged zeolites have the highest iodine adsorption capacities. Despite its inherent high cost, silver proved once again its ability to trap iodine compounds efficiently in an irreversible way. Furthermore, silver has the ability to form thermodynamically and chemically stable compounds with iodine.

### 2.3. Titanosilicates

Titanosilicates are a family that is analogous to aluminosilicate zeolites in which aluminium is replaced by titanium. Recently, Wu *et al.*^83^ studied silver-exchanged titanosilicates (named as ETS-10 and ETS-2) for trapping iodine (I_2_). The ETS-10 adsorbent is characterized by a stable chemical structure (isolated octahedral titanium chains in the silica network) with pores exclusively based on silicon. The composition of ETS-10 endows this adsorbent with hydrophobic properties and high chemical stability in acidic conditions. In contrast, ETS-2 is composed of sodium and titanium and possesses a reasonable specific surface area (260 m^2^ g^−1^), which corresponds to its external surface, without structural microporosity. Unlike ETS-10, ETS-2 has a high capacity for cation exchange (with sodium), like that of zeolites. It was shown that the iodine adsorption capacities of silver-doped and silver-exchanged titanosilicates were elevated, at around 220 g_I_2__ kg_adsorbent_^−1^ and 243 g_I_2__ kg_adsorbent_^−1^ in dry conditions for ETS-10 (35 wt% Ag) and ETS-2 (40 wt% Ag), respectively. The effect of humidity on the adsorption performance of these materials was also studied. A non-negligible decline of 30% in the adsorption capacity was measured for the Ag-ETS-10 adsorbent. According to the authors, the porous structure of the ETS-10 adsorbent favours the condensation of water inside the pores, which limits the accessibility of the silver adsorption sites for iodine. In the case of the non-porous ETS-2 adsorbent, the performance was much less affected by the presence of water. ETS-10 titanosilicate-type adsorbents supported on a hollow carbon nanostructured polyhedral adsorbent (C@ETS-10) were also studied.^94^ An adsorption capacity of 40 g_I_2__ kg_adsorbent_^−1^ at 20 °C was recorded.

### 2.4. Porous oxide materials

#### 2.4.1. Silver-doped silica and alumina (Ag/SiO_2_, Ag/Al_2_O_3_)

Silver-exchanged zeolites are not the only inorganic materials that are suitable for the capture of iodine compounds. Oxide-type materials such as alumina (Al_2_O_3_) and silica (SiO_2_) that are impregnated with silver, which are designated as Ag/Al_2_O_3_ and Ag/SiO_2_, respectively, are another class of adsorbents that can be found in the literature. These materials have been the focus of various studies^19,35,52,61,65,70,87–97^ and have been used for the capture of iodine (I_2_) on an industrial scale.^65,88–90,96^ These adsorbents, which contain silver nitrate (AgNO_3_), proved their efficiency for the elimination of iodine (I_2_) and iodomethane (CH_3_I) in a spent fuel reprocessing plant. The mechanism of the capture of iodine by these silver-impregnated materials has been detailed by Wilhelm *et al.*^48,54^ Silver nitrate (AgNO_3_) reacts with elemental iodine to form stable silver iodide (AgI) or silver iodate (AgIO_3_) by the following reactions:

14 AgNO_3_ + I_2_ → AgI + INO_3_

15 2INO_3_ + AgNO_3_ → AgIO_3_ + 3NO_2_ + 0.5I_2_

16 INO_3_ → NO_2_ + 0.5O_2_ + 0.5I_2_

17 AgNO_3_ + CH_3_I → CH_3_NO_3_ + AgI

An amorphous silver-doped silica, which was commercialized under the name of AC-6120, was used in the Karlsruhe spent fuel reprocessing plant (WAK) in Germany for the capture of iodine (I_2_).^88^ This adsorbent has a nominal BET surface area of 65–110 m^2^ g^−1^, a pore size distribution of 20–40 nm, a pore volume of 0.6 cm^3^ g^−1^ and a silver loading of 8–12 wt%. Decontamination factors (DFs) for iodine of between 100 (>99.0% efficiency) and 50 (98.0% efficiency) were achieved during its utilization on an industrial scale.^48,54,88^ On the laboratory scale, this silver-doped silica displayed decontamination factors for I_2_ that were higher than 10^4^ (>99.99% efficiency) at a temperature of 150 °C (bed length of 10 cm, superficial velocity of 25 cm s^−1^, in the presence of 1–5% NO_2_).^48,54^ The efficiency declined from 99.9944% to 27% at a relative humidity of 70% and 100%, respectively. In addition, Wilhelm and Schuttelkopf^48^ stated that an increase in temperature up to 200 °C slightly improved the performance. Nevertheless, an increase in temperature becomes less attractive in terms of balancing cost against efficiency.

On the other hand, Herrmann *et al.*^89^ studied alumina materials that were impregnated with silver (10 wt% Ag and 24 wt% Ag), which were developed in Japan at the Tokai spent fuel reprocessing plant^87,192^ for the retention of iodine species (I_2_ and CH_3_I). The silver-doped alumina was tested at 150 °C with a superficial velocity of 20 cm s^−1^ and an inlet concentration of CH_3_I of 30 ppm. A decontamination factor (DF) of higher than 500 (which corresponds to an adsorption capacity of 120 mg_CH_3_I_ cm_Ag/Al_2_O_3__^−3^) was achieved using alumina with 10 wt% Ag. For the alumina with the higher loading (24 wt% Ag), NO_*x*_ (1.5%) were introduced into the feed. In this case the decontamination factor exceeded 500 and corresponded to an adsorption capacity of 350 mg_CH_3_I_ cm_Ag/Al_2_O_3__^−3^. Therefore, this study demonstrated that the silver-doped adsorbents exhibited rather good resistance in the presence of NO_*x*_. In addition, it was reported^16^ that an elevated concentration of NO_2_ (between 1 and 10%) improved the iodine adsorption performance by preventing the reduction of silver (in the form of AgNO_3_) to metallic silver Ag^0^. The adsorption efficiency was about 99.9961% and 99.9973% in the presence of 1% and 10% NO_2_, respectively, in contrast to 99.9944% in the absence of NO_*x*_. It was also shown that a large quantity of organic contaminants, as well as a high relative humidity (>70%), could impair the performance of the material in the absence of a sufficient quantity of NO_*x*_.

Finally, the authors highlighted the fact that these types of adsorbents (Ag/Al_2_O_3_ and Ag/SiO_2_) were generally less expensive by a factor of 3 to 10 than silver-exchanged zeolites. Furthermore, an atmosphere of NO_2_ is needed to prevent the reduction of silver to its metallic form.

#### 2.4.2. Silver-doped mesoporous silica

Another class of materials, namely, mesoporous structured silica, has aroused the interest of scientists for iodine capture applications. These materials have been the focus of many studies, especially in the fields of catalysis and adsorption. Mesoporous silica materials are characterized by amorphous silica walls that delimit well-ordered mesocavities, a regular arrangement of mesopores and a particularly large specific surface area (it can reach values of higher than 1000 m^2^ g^−1^). These materials also possess the advantage of having pore sizes (3–10 nm) that are larger than those in zeolites (<1 nm), which ultimately improve the accessibility of the pores and active sites in mesoporous silica. Furthermore, silanol functions, which are localized on the silica surface, can be replaced *via* functionalization with organic and/or organometallic functional groups to improve their properties and performance depending on the intended applications. Few studies have focused on these materials for iodine capture applications.^81,84,86^ Mainly, the works by Mnasri *et al.* on silver-doped MCM-41 mesoporous silica,^84^ Yang *et al.* on bismuth-doped SBA-15^86^ and Chebbi on silver-doped SBA-15^81^ can be found in the literature.

The study by Mnasri *et al.*^84^ focused on the adsorption of iodine compounds by three types of silver-impregnated MCM-41 mesoporous silica with the following pore diameters: 2.4, 3.3 and 3.8 nm. Silver that was introduced into the mesoporous silica was reduced by NaBH_4_ to obtain metallic silver (between 1.65 and 2.16 wt% Ag). These materials were tested for the adsorption of iodine (I_2_) at 35 °C in the gas phase. The iodine adsorption capacities of the non-impregnated materials were on average between 90 and 130 g_I_2__ kg_adsorbent_^−1^. The difference in performance between the non-impregnated materials was associated with the discrepancy in their pore sizes and specific surface areas.^84^ In the presence of silver, the performance was significantly improved as the adsorption capacity reached 760–770 g_I_2__ kg_adsorbent_^−1^. The irreversibility of the capture of iodine at 120 °C and 527 °C was also studied by TGA, and no weight losses were indicated. In summary, these forms of mesoporous silica possess interesting and important iodine adsorption capacities, with the addition of a high degree of irreversibility when silver is added.

More recently, Chebbi^81^ studied, in parallel to his academic works on silver-exchanged zeolites, silver-doped mesoporous silica (SBA-15) for use in the field of iodine adsorption. Tests on the retention of CH_3_I ([CH_3_I] = 450 ppm, *T* = 10 °C) in the liquid phase were carried out on SBA-15 mesoporous silica doped with 0, 10, 20, and 40 wt% silver. Silver is assumed to be in the form of Ag^0^ nanoparticles in the mesopores and on the external surfaces with an average crystallite size of 20 nm. In the case of the non-impregnated parent compound SBA-15, an adsorption capacity of 49 g_CH_3_I_ kg_adsorbent_^−1^ was achieved. For the latter material, the capture of CH_3_I is controlled by physisorption-type interactions and is consequently reversible. The presence of Ag^0^ nanoparticles at different loadings inside the SBA-15 material significantly improves its iodine adsorption performance. Chebbi also highlights the quasi-linear relationship between the silver loading and the maximum amount of CH_3_I that can be adsorbed in the SBA-15. However, only a fraction of the silver is used to trap iodine. In fact, if the contribution of the physisorbed fraction of CH_3_I in the parent SBA-15 is considered (49 g_CH_3_I_ kg_adsorbent_^−1^ for 0 wt% silver), the I/Ag ratio in the silver-impregnated SBA-15 is particularly low (0.21 and 0.13 for a silver loading of 10 and 40 wt%, respectively). These results were compared with those for a silver-exchanged AgY zeolite in another work (silver loading of 23 wt%, Si/Al ratio of 40), in which the I/Ag ratio was 0.30 for a crystallite size of 9.1 nm. According to the authors, these discrepancies between the performance of silver-doped mesoporous silica and silver-exchanged zeolites mainly arise from the high dispersion of silver inside the structure. In addition, the absence of exchange sites in mesoporous silica and the large pore diameters facilitate the migration of silver to the surface, which leads to the aggregation of silver in the form of metallic nanoparticles, which ultimately block access to the pores. In the case of nanoparticles, only a small part of the silver can be used for the capture of iodine, which is probably due to the unfavourable core/shell ratio. In a similar way, the adsorption of I_2_ by the same silver-loaded SBA-15 ([I_2_] = 400 ppm, *T* = 25 °C) was studied.^81^ In another study by Chebbi,^81^ in the case of the parent compound SBA-15 no adsorption of iodine was observed. Once more, the presence of silver significantly improved the iodine adsorption performance. It should be noted that the efficiency of the capture of I_2_ per silver atom is higher than in the case of CH_3_I. A decrease in the efficiency of silver from I/Ag = 0.90 to I/Ag = 0.59 was also observed when the silver loading was increased from 10 to 40 wt%. In a similar way to zeolites with a high loading of silver, the less efficient utilization of silver can essentially be attributed to accessibility problems that prevented iodine from reaching silver inside pores.

#### 2.4.3. Other porous oxide materials

SBA-15 that was functionalized with aminopropyltrimethoxysilane (APTMS) was also used for the retention of iodine (I_2_) in the same standard conditions ([I_2_] = 400 ppm, *T* = 25 °C) as in previous studies by Chebbi.^81^ An iodine adsorption capacity of 179 g_I_2__ kg_adsorbent_^−1^ was achieved with this material. The affinity between I_2_ and the APTMS-functionalized silica was explained by the formation of a charge transfer complex. Then, this material was studied in conditions ([I_2_] > 600 ppm, *T* = 25 °C) such that the saturation capacity for iodine (600 g_I_2__ kg_adsorbent_^−1^) of the functionalized SBA-15 was reached. However, it should be noted that the functionalization with APTMS can deteriorate upon an increase in the temperature, and therefore it would not be efficient to use it for the adsorption of iodine in a nuclear power plant in the event of a severe nuclear accident.

In a study by Yang *et al.*,^86^ the remarkable efficiency for iodine capture of bismuth-doped mesoporous silica (SBA-15) was proved. Bismuth was incorporated into the SBA-15 material by previously modifying the silica surface with thiol groups and subsequent thermal treatment that led to the formation of a BI_2_S_3_ phase. The bismuth-doped mesoporous silica displayed a maximum iodine adsorption capacity of around 540 g_I_2__ kg_adsorbent_^−1^ (*T* = 200 °C, 6 h under static air). The good adsorption performance of the material was attributed by the authors to the strong interactions between bismuth sulphide and iodine, the elevated specific surface area and the porosity of SBA-15. Bismuth, when combined with iodine, can form particularly thermodynamically stable compounds such as BiOI and BiI_3_ (Δ_f_*G*° = −219.5 and −139.7 kJ mol^−1^, respectively). The large pores in SBA-15 (ranging from 50 to 300 Å, with a uniform distribution), as well as the strong affinity of bismuth for iodine, minimized the physical adsorption of iodine, which allowed chemisorption-type interactions to be the main capture process. Finally, the authors highlighted the low cost of bismuth and the easy preparation of SBA-15 as definite advantages for industrial applications. Another study led by Yang *et al.*^193^ on bismuth-based adsorbents (bismuth oxide) demonstrated an iodine trapping capacity that was approximately 1.9 times higher (0.468 g_I_2__ kg_adsorbent_^−1^) than that of commercial AgX zeolite.

Other porous oxide materials, such as metallic oxides (MgO, ZnO, La_2_O_3_, ZrO_2_, *etc.*) have been studied for trapping iodine in the gas phase.^194–197^ Among these, Glinski *et al.*^194^ reported that La_2_O_3_ and MgO had high iodine adsorption capacities of 31 and 48 g_I_2__ kg_adsorbent_^−1^, respectively, in comparison with other metal oxides at *T* = 100 °C under an anhydrous stream of N_2_ saturated with I_2_. More recently, Nandanwar *et al.*^195^ achieved a maximum iodine adsorption capacity of 196 g_I_2__ kg_adsorbent_^−1^ at room temperature with porous microspheres of magnesium oxide (the size of the microspheres was 5–7 μm).

Besides, no studies have focused on the utilization of all these mesoporous materials in conditions closer to those of real utilization (temperature, presence of inhibitors such as NO_*x*_ and H_2_O).

### 2.5. Silver-functionalized silica aerogels

**Fig. 9:**
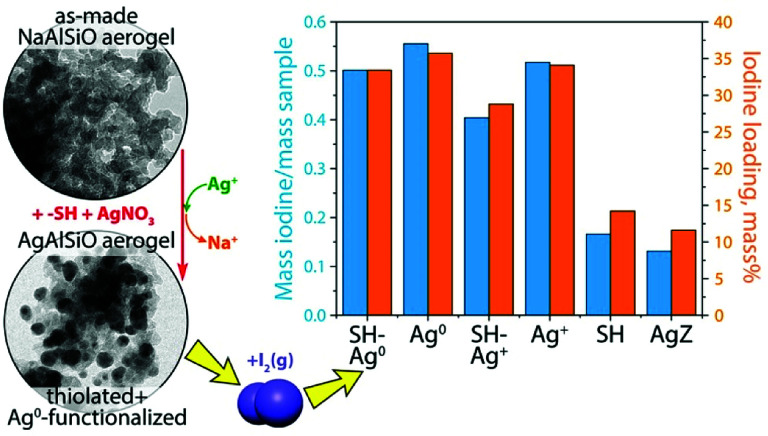
Comparison of the iodine adsorption capacities of different silver-functionalized aerogels (SH-Ag^0^, Ag^0^, SH-Ag^+^, Ag^+^, and SH) with that of a silver-loaded mordenite (AgZ).^105^ Reprinted with permission from B. J. Riley, J. O. Kroll, J. A. Peterson, J. Matyáš, M. J. Olszta, X. Li and J. D. Vienna, *ACS Applied Materials & Interfaces*, 2017, **9**, 32907–32919. Copyright 2017 American Chemical Society.

Several authors have studied aerogels as potential adsorbents for iodine trapping. Aerogels are porous materials that possess an elevated specific surface area (>1000 m^2^ g^−1^). Matyáš *et al.*^100–102^ developed silver-functionalized aerogels for trapping iodine compounds. These materials exhibited promising results, with an iodine adsorption capacity of 310 g_I_2__ kg_adsorbent_^−1^ and a decontamination factor (DF) of higher than 10^5^. More recently, Riley *et al.*^105^ studied silver-functionalized aluminosilicate aerogels (Na–Al–Si–O) for the capture of iodine. An iodine adsorption capacity of greater than 500 mg_I_2__ g_adsorbent_^−1^ was observed. For comparison, the adsorption capacity of silver-exchanged mordenite (Ag^0^MOR) tested in the same conditions only reached 190 mg_I_2__ g_adsorbent_^−1^ (Fig. 9). These studies proved that silver-functionalized aerogels can be potential candidates for iodine capture. However, few articles on the subject can be found in the literature and no test was performed in conditions similar to those of real utilization.

### 2.6. Chalcogen-based aerogels (chalcogels)

Aerogels based on chalcogens (S, Se, Te), which are called “chalcogels”, have been the focal point of a few studies on iodine trapping.^8,85,90,91,93–95^ Chalcogels are synthesized from chalcogen clusters (Ge_4_S_10_^4−^, Sn_2_S_6_^4−^, Sn_4_S_4_^4−^, MoS_4_^4−^) interconnected by a secondary metallic species (such as Pt^2+^, Co^2+^, Sn^2+^, Sb^3+^, Bi^3+^, Ni^2+^, and Zn^2+^). Chalcogens enclosed in chalcogels are classified as weak Lewis bases according to the HSAB concept (hard and soft acids and bases). Consequently, they have a high affinity for iodine (I_2_), which is known to be a weak Lewis acid. This strong affinity for iodine was demonstrated for a large range of chalcogels, including PtGe_2_S_5_, Sn_2_S_3_, CoMoS_4_, NiMoS_4_, CoS_5_, Sb_4_(SnS_4_)_3_ and ZnSn_2_S_6_.^7,104,107,108^ Riley *et al.*^107^ developed SnS (Sn_2_S_3_) structured chalcogels for trapping iodine (I_2_) with an iodine uptake of up to 32.7 and 68.3 wt% for the best chalcogel formulations. Furthermore, the adsorption mechanism was studied. During adsorption, iodine interacts with SnS-type chalcogels by chemical reactions (chemisorption) to form crystalline SnI_4_ and SnI_4_(S_8_)_2_ species, which was observed by X-ray diffraction analysis. This reaction is particularly favourable, with a Gibbs formation enthalpy of Δ_f_*G*° = −215.1 kJ mol^−1^ at 298.15 K (25 °C). Subrahmanyam *et al.*^7^ carried out studies of the adsorption of iodine onto NiMoS_4_, CoMoS_4_, Sb_4_Sn_3_S_12_, Zn_2_Sn_2_S_6_ and K_0.16_CoS_*x*_ (*x* = 4–5) chalcogel-type adsorbents. A mass uptake of up to 225 wt% was achieved for the Zn_2_Sn_2_S_6_ and NiMoS_4_ chalcogels. As already mentioned, this high iodine adsorption capacity is due to the chemical reaction between iodine and the adsorbent (by chemisorption) and also the large specific surface areas that range from 200 to 490 m^2^ g^−1^. Iodine reacts chemically with Zn_2_Sn_2_S_6_, Sb_4_Sn_3_S_12_ and K_0.16_CoS_*x*_ (*x* = 4−5) chalcogels to form metallic halides such as SnI_4_, SbI_3_ and KI, whereas only physisorption interactions are observed for NiMoS_4_ and CoMoS_4_ chalcogels. TGA analysis^7^ showed that Zn_2_Sn_2_S_6_ and Sb_4_Sn_3_S_12_ chalcogels with stored iodine were stable up to 150 °C, but beyond that temperature iodine was released progressively (40 wt% in 30 days). Moreover, the other chalcogels release iodine above 75 °C. The weight loss is attributed to the sublimation of iodine molecules and SnI_4_, as well as the release of physisorbed iodine from the surface. In conclusion, this type of material possesses excellent iodine adsorption capacities. However, the thermal stability of iodine capture (or the formed iodides) is quite low.

### 2.7. Macroreticular resins

Synthetic macroreticular resins are macromolecular structures formed by polymerisation reactions (from vinylbenzene monomers, for example). Functionalized chemical groups, such as acrylic esters and polystyrene, can be grafted onto the structural framework of the resins. A resin can be defined by the following properties: its crosslinking rate (percentage of crosslinking monomer in the resin), the porosity of the framework (presence of variable pore and/or channel sizes), its granulometric composition (mean size of resin beads), its selectivity and its ion exchange capacity (quantity of ions that can be fixed for a given mass or volume of the resin). In general, macroreticular resins possess elevated specific surface areas (up to 900 m^2^ g^−1^) and can exhibit cation or anion exchange properties. Resins are known to have high chemical stability and can be restored to their initial state by washing with the appropriate solutions. Furthermore, their crosslinked structure does not limit the diffusion of molecules or substances and therefore helps to increase their storage and/or trapping capacities. The possibility of employing these kinds of adsorbents for trapping iodine has been the focus of several studies.^16,39,41,42,44–46,50,53,55^ However, very few studies have focused on the mechanism of the trapping of iodine (I_2_) and organic iodine compounds by these adsorbents. In general, macroreticular resins have a strong affinity for any kind of substance thanks to their tunable hydrophobic and hydrophilic properties. Because most of these adsorbents also have non-ionic functions, they can have a strong affinity for uncharged molecules such as iodine (I_2_) or organic iodine compounds such as CH_3_I. Most studies focused on the trapping of iodine in aqueous solutions. Few studies have focused on the behaviour of these adsorbents in the gaseous phase for iodine adsorption applications because of their limited stability under air. The objective was to develop an iodine filtration medium that was both cheaper than silver-doped adsorbents (*e.g.*, silver-exchanged zeolites) and more stable than activated carbons.^16^ In 1968, Hirling^39^ studied a cation exchange resin (Varion KS) for iodine trapping applications. Tests showed a strong affinity for iodine with adsorption capacities of between 872 and 1437 g_I_2__ kg_adsorbent_^−1^. However, because of the saturation of the adsorbent bed, an efficiency of only 40% was achieved. This resin was also impregnated with silver to capture HI, which endowed the resin with a trapping capacity of 93.1% and an HI adsorption capacity of 1437 g_HI_ kg_adsorbent_^−1^. Resins of another type, namely, Amberlite XAD, were also tested for trapping iodine compounds. These resins are macroporous and can have rigid and three-dimensional structures, depending on the monomer used. These adsorbents can incorporate a large quantity of extractant thanks to their elevated specific surface areas, high mechanical resistance, superior crosslinking and low swelling during impregnation. Amberlite XAD resins have specific surface areas of between 150 and 900 m^2^ g^−1^ with a mean pore diameter of between 4 and 9 nm and a pore volume of 0.6 to 1.1 cm^3^ g^−1^. The first studies were carried out by Moore *et al.*^41,42,44,46^ At 21 °C in dry conditions, decontamination factors (DFs) of higher than 10^4^ were achieved for an iodine (I_2_) adsorption capacity of 213 mg_I_2__ g_adsorbent_^−1^. With Amberlite XAD-12 resin, a maximum iodine adsorption capacity of 1.39 g_I_2__ g_adsorbent_^−1^ at 25 °C was achieved, but only 84 mg_I_2__ g_adsorbent_^−1^ could be adsorbed at 50 °C. In the same way, maximum iodine adsorption capacities of 278 mg_I_2__ g_adsorbent_^−1^ and 15 mg_I_2__ g_adsorbent_^−1^ at 21 °C and 25 °C, respectively, were achieved with Amberlite XAD-4 resin. However, it is quite surprising to observe such a great discrepancy for a temperature difference of 4 °C. At higher temperatures and in the presence of humidity a decrease in the decontamination factor was observed. In conclusion, macroreticular resins possess elevated iodine adsorption capacities (between 200 and 1437 mg g_adsorbent_^−1^).^41,42,44,46^ Furthermore, these materials are fairly stable in the presence of irradiation.^16,50,55^ On the other hand, significant decreases in their adsorption capacities were observed at high temperatures (>50 °C) or in the presence of humidity. These drawbacks would drastically limit their utility in the real conditions of a severe nuclear accident.

### 2.8. Metal–organic frameworks (MOFs)

In the past fifteen years, the number of studies on metal–organic frameworks (MOFs) has grown sharply thanks to their very large specific surface areas (up to ∼10^4^ m^2^ g^−1^) and well-defined pore sizes.^8^ MOFs are organic–inorganic hybrid materials that consist of inorganic parts comprising metal ions, which are referred to as secondary building units, linked by organic entities, which are most commonly referred to as ligands. The secondary building units can be composed of single metallic cations, dimers, trimers, chains, planes and/or a three-dimensional structure. The organic ligands need to possess a charge and/or unbound electron pairs, which ensure strong bonds with the inorganic components of the framework. The most commonly used ligands are organic compounds that contain carboxylic acid or imidazole (C_3_H_4_N_2_) groups. The best advantages of these types of materials are their large assortment of organic and inorganic entities that can generate a wide range of MOFs with various structures, topologies and pore sizes. In 2003, Abrahams *et al.*^121^ demonstrated the feasibility of introducing iodine into a hydrated zinc saccharate ([Zn(C_6_H_8_O_8_)]·2H_2_O). Since then, MOFs have been particularly popular for the retention of iodine.^6,14,109,110,115,116,118–127,129–132^ Most of the studies were carried out on materials based on divalent metals such as Zn^2+^ and Cu^2+^, as well as aluminium-based structures. In fact, the most commonly studied MOFs for the capture of iodine were the zeolitic imidazolate framework Zn(2-methylimidazolate)_2_ (ZIF-8) and HKUST-1 (Cu_3_(benzene-1,3,5-tricarboxylate)_2_(H_2_O)_3_, Cu-BTC).^14,115,124,125,129,132^ These two MOFs have elevated specific surface areas of 1875 and 1798 m^2^ g^−1^, respectively, with pore diameters of 11.6 and 9 Å, respectively.^127^ The most prominent studies on this subject were carried out by Sava *et al.*^14,124,125,129,130^ In fact, Sava *et al.*^14^ performed experiments on the iodine capture capacity of ZIF-8 at atmospheric pressure with a vapour pressure of iodine (I_2_) of 0.014 atm at 350 K. Once equilibrium had been reached (5–12 hours), the iodine adsorption capacity of ZIF-8 was 1.25 g_I_2__ g_adsorbent_^−1^ (1250 g_I_2__ kg_adsorbent_^−1^) for an I/Zn ratio of 2.2. According to the results from Sava *et al.*, the capture of iodine is due to the favourable interactions between iodine and the ligand (2-methylimidazolate, MeIM). Only 25% of the iodine is localized at the surface of the MOF, whereas 75% of the iodine is confined in the sodalite cages of ZIF-8. Studies by Hughes *et al.*^129^ give complementary information on the thermal stability of, and chemisorption of iodine by, ZIF-8. They showed that iodine interacts strongly with hydrogen atoms in the methyl group on the one hand and the carbon atom in the ligand methine (=CH–) group on the other hand. Furthermore, they observed that the complex formed inside the sodalite cages is thermally stable at temperatures of up to 300 °C, which is the limit of the thermal stability of the ZIF-8 material.^129^ However, iodine molecules that are adsorbed on the external surface interact weakly with the material (Δ*H*_ads_ = −18.06 ± 2.03 kJ mol^−1^), which ultimately permits the release of iodine at temperatures below 125 °C. On the other hand, iodine that is confined in the cages is thermally stable with an adsorption energy of Δ*H*_ads_ = −41.47 ± 0.62 kJ mol^−1^.

Another study by Sava *et al.*^130^ focused on the iodine adsorption capacity of Cu-BTC (HKUST-1). The study of this material was carried out at atmospheric pressure with a relative humidity of 3.5% and at 77 °C. Iodine was introduced in the vapour phase with a ratio to water of 1 : 1 (vapour pressures of I_2_ and water of 10.47 and 10.11 mmHg, respectively). An adsorption capacity of 1.75 g_I_2__ g_adsorbent_^−1^ (1750 g_I_2__ kg_adsorbent_^−1^) for an I/Cu ratio of 3 was found. *Via* this study, the authors demonstrated that the adsorption of iodine occurs in two steps. In the first step, iodine is adsorbed in the small triangular cages of the MOF (5 Å), and then interactions take place in the larger cages (11 and 13.55 Å) thanks to the strong interactions between iodine and the benzene rings of the tricarboxylate ligand (van der Waals interactions). Finally, in the larger cages, I_2_–I_2_ intermolecular interactions could also be observed. Furthermore, the favourable adsorption of iodine in preference to water (0.15 g_H_2_O_ g_adsorbent_^−1^) was explained by the formation of a hydrophobic barrier due to the presence of iodine (iodine limits the access of water to the adsorption sites), which makes this material very interesting for real applications, including severe nuclear accidents. However, high temperatures would still be harmful to this kind of material.

Aluminium-based MOFs have also been studied for iodine trapping applications.^115,116,120^ Studies were carried out on the MIL-53 family of MOFs. These materials are particularly attractive owing to their high chemical and thermal stability. Furthermore, these materials are commercially available. Falaise *et al.*^116^ studied seven aluminium-based MOFs of the MIL family (MIL-96, MIL-100, MIL-101-NH_2_, MIL-118, MIL-120, CAU-1, and MIL-53-X, where X is H, Cl, Br, CH_3_, NH_2_, NO_2_, (OH)_2_, COOH, or (COOH)_2_) with different structural properties (functionalization, pore diameter, specific surface area, *etc.*). They first studied the MIL-53-X family to examine the effect of functionalization on the iodine adsorption capacity in the liquid phase. Among the above functional groups (H, Cl, Br, CH_3_, NH_2_, NO_2_, (OH)_2_, COOH, and (COOH)_2_), only MOFs that contained electron-donating groups (NH_2_ and OH) exhibited interesting iodine capture properties. In the case of non-functionalized MIL-53, only 5% of iodine was adsorbed from a solution in cyclohexane after 48 hours. In contrast, the best performance was achieved with MIL-53-NH_2_, which exhibited a maximum iodine adsorption of 60% after 48 hours. For the other MOFs that were studied, only MIL-101-NH_2_ displayed excellent iodine adsorption capacities, with an efficiency of 90% after only 30 hours. Other MOFs such as CAU-1 and MIL-120 also exhibited fairly good performance (∼80% after 30 hours). In the same context, Assaad *et al.*^120^ confirmed that MIL-101 could exhibit an iodine adsorption efficiency of higher than 90% (96.61%).

Some computer simulation studies on the adsorption of iodine on MOFs have been performed. Assfour *et al.*^119^ studied the performance of twelve MOFs for the retention of iodine by molecular modeling. Their simulations obtained definite information and an understanding of the influence of the pore volumes and specific surface areas on the iodine storage capacity. They showed that MOFs with elevated pore volumes and specific surface areas were most suitable for the capture of iodine in conditions of normal temperature and pressure. However, at lower pressures MOFs with the smallest pore volumes were more suitable for the capture of iodine. Nevertheless, several materials exhibited important iodine adsorption capacities in conditions of normal temperature and pressure (up to ∼13 g_I_2__ g_adsorbent_^−1^ for NU-110). The modelling studies by Yuan *et al.*^125^ helped to identify ZIF-10 as the MOF with the most important iodine adsorption capacity (2.39 g_I_2__ g_adsorbent_^−1^ at 25 °C and in conditions of moderate pressure).

In conclusion, the studies in the literature mainly focused on the adsorption capacities of MOFs for iodine (I_2_), whereas no studies have focused on organic iodine compounds such as CH_3_I. MOFs with high iodine capacities are a promising alternative for the capture of iodine, principally thanks to their high adsorption capacities. Furthermore, in the case of Cu-BTC, iodine is preferentially adsorbed rather than water, which is a major advantage in the real conditions of a severe nuclear incident. However, these materials have limited thermal stability and are not produced on a large scale, which makes them particularly expensive.

### 2.9. Covalently linked porous organic polymers (POPs)

In parallel to the increasing development of MOFs, porous organic polymers (POPs) have recently attracted attention owing to their highly tunable molecular design, large surface areas, low skeleton densities (lightweight elements), strong covalent linkages, high physicochemical stabilities and tunable porosities.^133–143^ Although POPs are usually amorphous solids, unlike crystalline MOFs, their excellent physicochemical stability makes them more suited for real applications. In addition, the synthetic diversity of POPs makes it possible to attain control over their functionality by the rational design and choice of molecular building blocks. On the basis of their physicochemical robustness and pore features, POPs have huge potential to be efficient adsorbents for the capture of volatile radioactive iodine. It is widely accepted that the introduction of active sites such as metallic species, N, S, and C=C into the porous network of POPs can greatly improve their affinity for iodine molecules, which ultimately significantly increases the uptake of iodine. In general, the uptake of iodine depends on both the pore features (pore size and/or pore volume) and the affinity of the adsorbent for iodine molecules. To date, a certain number of POPs have been employed for this purpose, such as nanoporous organic polymers (NOPs),^134^ conjugated microporous polymers (CMPs)^139,141,142^ such as metalloporphyrin-based CMPs^138^ and hexaphenylbenzene-based CMPs,^135^ azo-bridged porous triptycene networks^140,143^ and crystalline covalent organic frameworks (COFs).^111–114,117,128^

Chen *et al.*^134^ studied the iodine adsorption performance of a series of hierarchically porous organic polymers based on tetraphenyladamantane (named as NOP-53, NOP-54 and NOP-55). Their pore properties were controlled by adjusting the lengths and rigidities of the linkers; for example, rigid tetrahedral building blocks of 1,3,5,7-tetraphenyladamantane were linked with flexible alkyl chains to obtain different hierarchical NOPs. NOP-53 and NOP-54 feature hierarchically porous structures, whereas NOP-55 was found to be only microporous with a broad micropore size distribution. NOP-54 exhibits the largest BET specific surface area and pore volume (1178 m^2^ g^−1^ and 1.32 cm^3^ g^−1^, respectively), followed by NOP-53 (744 m^2^ g^−1^ and 0.73 cm^3^ g^−1^, respectively) and NOP-55 (526 m^2^ g^−1^ and 0.42 cm^3^ g^−1^, respectively). Chen *et al.*^134^ determined their uptakes of iodine (I_2_) by gravimetric measurements. Samples were loaded into a sealed container in the presence of iodine pellets. The container was degassed and kept at 75 °C. According to Chen *et al.*,^134^ the iodine uptake increased gradually over a period of 4 hours until the system reached saturation. NOP-54 displayed the highest uptake of iodine of up to 2.02 g_I_2__ g_adsorbent_^−1^, followed by NOP-53 (1.77 g_I_2__ g_adsorbent_^−1^) and NOP-55 (1.39 g_I_2__ g_adsorbent_^−1^). According to the authors, NOP-53 and NOP-54 adsorb iodine at a faster rate than NOP-55. They attributed these observations to the existence of the hierarchical porous structures, which facilitated the transport of iodine in the networks.

Li *et al.*^143^ developed in 2016 a novel porous azo-bridged porphyrin–phthalocyanine network, which was synthesized by combining an azo skeleton with π-conjugated building blocks. AzoPPN was synthesized by a catalyst-free coupling reaction between the free base form of 5,10,15,20-tetrakis(4-nitrophenyl)porphyrin (H_2_TPP(NO_2_)_4_) and nickel tetraaminophthalocyanine (NiPc(NH_2_)_4_) under alkaline conditions. This adsorbent has a BET specific surface area of 400 m^2^ g^−1^. According to the authors, the porous structure, together with the porphyrin and phthalocyanine units, provides effective sorption sites that can greatly increase the affinity for iodine both physically and chemically. As a result, AzoPPN exhibits an iodine adsorption capacity of up to 2.9 g_I_2__ g_adsorbent_^−1^. Similarly, Dang *et al.*^140^ studied an azo-linked porous organic network (Azo-Trip), in which triptycene was incorporated as a building block *via* a facile Zn-induced reductive homocoupling reaction. This adsorbent also has a large BET surface area (510 m^2^ g^−1^) and an iodine uptake of up to 2.38 g_I_2__ g_adsorbent_^−1^.

Conjugated microporous polymers (CMPs) are a class of amorphous materials that permit the linking of building blocks in a π-conjugated fashion and possess three-dimensional (3D) networks. They are usually synthesized using metal-catalyzed cross-coupling chemistry to form 3D networks with extended π-conjugation. Their finely tuned porosity, very large specific surface area, and relatively high thermal and chemical stability, which originates from their rigid π-conjugated structure, and the high affinity of I_2_ for π-conjugated CMP networks make them attractive candidates for the adsorption and capture of radioactive iodine molecules.

Sigen *et al.*^138^ developed a new conjugated microporous polymer based on a metalloporphyrin (NiP-CMP), which was synthesized *via* a homocoupling polymerization reaction. NiP-CMP possesses a large BET surface area of greater than 2600 m^2^ g^−1^ and a large pore volume of 2.3 cm^3^ g^−1^. To determine its iodine adsorption performance, the NiP-CMP material was placed in a sealed vessel in the presence of solid iodine. The iodine sublimed into the porous adsorbent over time at 77 °C and ambient pressure, which are typical conditions in fuel reprocessing. An uptake of iodine (I_2_) of 2.02 g_I_2__ g_adsorbent_^−1^ from iodine vapour was determined by gravimetric measurements. Similarly, Chen *et al.*^139^ studied conjugated microporous polymer nanotubes (CMPNs) for the capture of iodine. The material exhibited a maximum uptake of 2.08 g_I_2__ g_adsorbent_^−1^ in the adsorption of I_2_. Sigen *et al.*^138^ also studied the reversibility of adsorption and regeneration of the sorbent. In fact, the authors easily removed iodine from the framework by immersing the I_2_@NiP-CMP material in an organic solvent (96% of iodine was desorbed after 8 hours in ethanol). The authors highlighted the fact that the absorbed iodine can be recovered and that the NiP-CMP sorbent can be easily recycled and reused. The reversibility of the material was also confirmed for the CMPN material studied by Chen *et al.*^139^

Ren *et al.*^141^ studied the iodine adsorption performance of conjugated microporous polymers (SCMPs). Two SCMP networks (SCMP-I and SCMP-II), which are based on the monomer 3,3′,5,5′-tetrabromo-2,2′-bithiophene and were obtained by a palladium-catalyzed Sonogashira–Hagihara cross-coupling reaction, have interesting honeycomb-like porous 3D network structures. SCMP-I is composed of agglomerated spheres with different sizes, whereas SCMP-II has an intertwining porous structure. Furthermore, the specific surface areas were found to be 2.72 m^2^ g^−1^ for SCMP-I and 119.76 m^2^ g^−1^ for SCMP-II. The uptakes of iodine (I_2_) were determined by gravimetric measurements, and the capture of iodine vapour was conducted at 80 °C and ambient pressure. The authors suggest that the high uptake of iodine by SCMP-II of up to 3.45 g_I_2__ g_adsorbent_^−1^ may be attributed to its unique macroscopically honeycomb-like porous features, as well as the π-conjugated network structure, which has been confirmed to have a relatively strong affinity for iodine molecules.

Liao *et al.*^135^ studied a series of conjugated microporous polymers based on hexaphenylbenzene (HCMPs) with secondary amine functional groups. The HCMPs had a moderate microporous BET surface area of up to 430 m^2^ g^−1^ and a narrow pore size distribution with a uniform ultramicropore size of less than 1 nm. These materials exhibit excellent iodine adsorption capacities with an uptake of iodine of up to 3.16 g_I_2__ g_adsorbent_^−1^, with the possibility of a further increase in the uptake to 3.36 g_I_2__ g_adsorbent_^−1^ when the polymers are reduced with anhydrous hydrazine. The authors studied the desorption rate of the iodine-loaded HCMPs by placing the materials in organic solvents such as ethanol at room temperature. Furthermore, Liao *et al.* showed that the release of iodine can be induced by heating the iodine-loaded material at 120–200 °C. An iodine-loaded HCMP was heated at 125 °C in air for 30 minutes. They achieved an iodine release efficiency of 98.8%. In addition, recycling of the HCMP material was studied, and the iodine uptake capacity was found to be 2.95 g_I_2__ g_adsorbent_^−1^ and 2.88 g_I_2__ g_adsorbent_^−1^ upon completion of the first and second cycles, respectively, which represented a retention of 93.3% and 91.3%, respectively, of the initial capacity. The authors emphasized the fact that these materials are attractive as robust, recyclable and reversible adsorbents for iodine uptake.

Zhu *et al.*^142^ developed two novel conjugated porous materials based on BODIPY for studies of the adsorption of iodine (I_2_). BDP-CPP-1 and BDP-CPP-2 were synthesized *via* a Sonogashira cross-coupling reaction between 1,3,5-triethynylbenzene (TEB) and dibromo-substituted derivatives. Both materials exhibit high iodine adsorption capacities of 2.83 g_I_2__ g_adsorbent_^−1^ and 2.23 g_I_2__ g_adsorbent_^−1^, respectively. Owing to the highly π-conjugated porous structure (coexistence of triple bonds, phenyl rings and aromatic pyrrole moieties) of BODIPY, BDP-CPP-1 displays a high capacity (2.83 g_I_2__ g_adsorbent_^−1^) for volatile iodine at 75 °C and a high iodine adsorption rate in an organic solution at 25 °C (90% of iodine in a solution in hexane was captured after 7 hours). According to the authors, these high capacities can be attributed to the large BET surface area (635 m^2^ g^−1^) and pore volume (0.78 cm^3^ g^−1^) and the chemical substitution reaction at the 2- and 6-positions of the BODIPY core (*i.e.*, adsorption of volatile iodine *via* a chemical mechanism involving the hydrogen atoms at the 2- and 6-positions). Furthermore, the BDP-CPPs display high thermal stability, with a decomposition temperature of about 300 °C. In addition, these CMPs exhibit excellent recyclability after 4 cycles, which, according to the authors, may result from the π-conjugated porous structure.

Finally, covalent–organic frameworks (COFs) are a class of material in which organic ligands are linked together to form a periodic structure *via* strong covalent bonds. COFs have a well-ordered architecture with particularly interesting structural properties, such as a low structural density, easily tuned porosity, elevated specific surface areas, and quite high thermal (up to 600 °C in the best case) and chemical stability, as well as a wide range of functionalization.^117,128,198^ Lan *et al.*^128^ used computational studies to determine the theoretical adsorption capacities of COFs for iodine (I_2_) and iodomethane (CH_3_I) in the conditions of real applications. The results show that 3D-COFs have the best adsorption performance for I_2_ and CH_3_I in contrast to 2D-COFs. A pyrene-based 3D-COF (3D-Py-COF) has been found to possess a high adsorption capacity for iodine compounds of 16.7 g_I_2__ g_adsorbent_^−1^, which is an extremely high value in comparison with those for the other adsorbents described in this review. The morphologies of the pores play a crucial role in the adsorption of CH_3_I. Consequently, 3D-COFs with a ctn topology and a pore size of 9 Å display the highest adsorption capacities in comparison with other COFs. COF-103 was identified as the best adsorbent for CH_3_I, with an adsorption capacity that reached 2.8 g_I_2__ g_adsorbent_^−1^. COFs have not yet been the focus of many studies.^111–114,117,128^ Furthermore, they are still new laboratory materials and are therefore produced in very small quantities at high prices.

## 3. Summary and conclusion

Taking into account the severe operating conditions in the case of a nuclear incident, sorbents for the capture of radioactive iodine (CH_3_I and I_2_) should fulfil the following requirements: a high adsorption capacity over a wide temperature range, irreversibility of capture, selectivity, high thermal stability of the adsorbed species, high stability against irradiation and strong resistance in the presence of molecules of various gases such as water and NO_*x*_ (Table 2). Various kinds of porous sorbents have been studied for the capture of iodine. Activated carbons doped with KI and TEDA and porous solids doped with silver (zeolites, porous silica and alumina, aerogels and porous titanosilicates) have been the most widely studied sorbents. Activated carbons proved to exhibit good performance in the adsorption of iodine, especially when loaded with KI and TEDA, with strong capture of radioactive iodine. However, their performance declined significantly in the presence of humidity (>40%) and at high temperatures (*T* > 80 °C), whereas such operating conditions are probable in the case of a severe nuclear accident. Furthermore, they are particularly affected by NO_*x*_ and aging. Silver-doped materials, in particular, silver-exchanged zeolites, were meant to overcome the issues with activated carbons by being more resistant to harsh conditions such as high humidity, the presence of NO_*x*_ and high temperatures. Most studies revealed that silver-exchanged zeolites exhibit high performance and capacities for the capture of iodine with strong irreversible adsorption of iodine. The best results were obtained for zeolites with large pores (AgX and AgY) and a high silver content. On the other hand, silver-based sorbents proved to be particularly expensive in comparison with activated carbons and other sorbents. The doping of zeolites with less expensive metals was found to be much less effective in comparison with silver. However, bismuth-doped mesoporous silica displays high capacities for the adsorption of iodine and thermal stability of the captured iodine.

**Table 2:** Summary of the advantages and drawbacks of the various porous sorbents described in this review

Adsorbents	Advantages	Drawbacks
Activated carbons	Good adsorption performance if impregnated with KI and/or TEDA (DF of up to 10^4^)	Decline in adsorption performance in the presence of humidity (>40%)
	Low production cost	Decline in adsorption performance at high temperatures (*T* > 80 °C)
	High stability in basic and acidic condition	Alteration of the adsorption performance due to aging
		Low autoignition temperature
		Strong influence of NO_*x*_ (formation of explosive compounds)
Silver-exchanged zeolites	Good adsorption performance (optimal adsorption temperature: 423 K)	Expensive
	Trapping by precipitation (formation of AgI) + high capture stability	Alteration of the adsorption performance due to organic compounds (especially chlorides)
	High irreversibility of the trapping of iodine (especially for AgY zeolite, >80%)	Negative effects of NO_*x*_ and humidity on performance
	No or little influence of γ-radiation	
	Tunable chemical and structural properties	
	High thermal and chemical stability	
Silver-doped silica and/or alumina (Ag/SiO_2_ and Ag/Al_2_O_3_)	Good adsorption performance (DF > 10^2^) up to 150 °C	Loss of efficiency at temperatures of <200 °C + higher operating cost
	In the presence of NO_*x*_ (1–10%), an increase in performance	Loss of performance in the presence of a large excess of organic contaminants
	Less expensive by a factor of 3 to 10 than silver-doped zeolites (theoretically)	Alteration of the adsorption performance with humidity (>70%)
Mesoporous silica	Adsorption performance similar to that of silver-doped zeolites	Few studies on the influence of humidity and/or inhibitors (NO_*x*_, organics, *etc.*)
	Elevated adsorption capacities (up to 0.6 g_I_2__ g_adsorbent_^−1^) when functionalized (*e.g.*, with APTMS)	Limited thermal stability when functionalized
Aerogels/chalcogels	Excellent adsorption performance (>0.5 g g_adsorbent_^−1^)	Few studies on the influence of humidity and/or inhibitors (NO_*x*_, organics, *etc.*)
		Little literature on these materials
		Expensive
Titanosilicates	Adsorption performance similar to that of silver-doped zeolites when doped with silver	Low adsorption capacities
		Few studies on the influence of humidity and/or inhibitors (NO_*x*_, organics, *etc.*)
Macroreticular resins	Excellent adsorption performance (up to 1 g g_adsorbent_^−1^)	Significant decline in adsorption performance at temperatures of higher than 50 °C or in the presence of humidity
	Stable in presence of γ-radiation	
	Resistant in acidic conditions (NO_*x*_)	
Metal–organic frameworks (MOFs)	Excellent adsorption performance (up to 1.75 g_I_2__ g_adsorbent_^−1^) theoretically and in ideal conditions	No studies on the influence of humidity and/or inhibitors (NO_*x*_, organics, *etc.*)
	High selectivity toward I_2_ rather than water	No information on the adsorption and capture of CH_3_I
	Trapping *via* chemisorption/physisorption	Low thermal stability at high temperatures
	Large range of existing MOFs (pores, specific surface areas, variable structures) + possibility of adjusting properties	Expensive
		Laboratory objects
Porous organic polymers	Excellent adsorption performance (up to 3.45 g_I_2__ g_adsorbent_^−1^)	No information on the adsorption and capture of CH_3_I
	Highly tunable molecular design	No studies on the influence of humidity and/or inhibitors (NO_*x*_, organics, *etc.*)
	Low skeleton densities (lightweight elements)	Expensive
	High physicochemical stabilities and tunable porosities reversible adsorption of iodine	Laboratory objects
Covalent–organic frameworks (COFs)	Excellent adsorption performance (up to 16.7 g_I_2__ g_adsorbent_^−1^) theoretically and in ideal conditions	Theoretical studies only
	Studies on the adsorption and capture of I_2_ and CH_3_I	No studies on the influence of humidity and/or inhibitors (NO_*x*_, organics, *etc.*)
	Large range of existing COFs (pores, specific surface areas, variable structures)	Little literature on these materials for the capture of iodine compounds
		Expensive
		Laboratory objects

Interesting results have also been obtained for chalcogen-based aerogels, in particular, those based on tin sulphide, but the thermal stability of the captured iodine is relatively low and the influence of humidity on the capture of iodine has not yet been studied. Other porous sorbents such as metal–organic frameworks (MOFs) and porous organic polymers (POPs) demonstrated great potential for the trapping of iodine. All these sorbents possess good iodine adsorption capacities, which reach 1750 g_I_2__ kg_adsorbent_^−1^ in the case of MOFs. However, although they have promising iodine adsorption properties, most of these sorbents were not fully characterized, and more precise studies on the influence of various operating parameters (such as temperature, humidity, and the presence of NO_*x*_) are still needed before an accurate judgement regarding the best sorbent for iodine removal applications. Furthermore, some sorbents, such as MOFs and POPs, are laboratory objects and thus not suitable for industrial applications at the moment. It can be concluded that these new sorbents should be studied more thoroughly from the point of view of real applications, which implies cost-effective solutions and tests in a wide range of operating conditions.

Thus, the development of new sorbents that combine a high iodine adsorption capacity over a wide temperature range, high stability and selectivity in the capture of iodine at high temperatures and humidities and in the presence of other molecules, and low cost still remains a challenge. The use of several kinds of iodine sorbents to combine their advantages could be a promising way to fulfil the requirements of real applications.

## Conflicts of interest

There are no conflicts to declare.

## Supplementary Material

RA-008-C8RA04775H-s001
