# Supplementary material for: Porous sorbents for the capture of radioactive iodine compounds: a review
Source: RSC Adv. 2018 Aug 17;8(51):29248–73. doi: 10.1039/c8ra04775h (PMC9086542; doi:10.1039/c8ra04775h)
Supplement: RA-008-C8RA04775H-s001 [file RA-008-C8RA04775H-s001.pdf]

Electronic Supplementary Material (ESI) for RSC Advances.
This journal is © The Royal Society of Chemistry 2018

Supporting information

Porous sorbents for the capture of iodine radioactive compounds: A review

Joffrey Huve[a,b], Andrey Ryzhikov[a,b], Habiba Nouali[a,b], Virginie Lalia[c], Grégoire Augé[d] and T. Jean Daou[a,b]*

[a.] Université de Haute Alsace (UHA), Axe Matériaux à Porosité Contrôlée (MPC), CNRS IS2M UMR 7361, 3 bis rue Alfred Werner, F-68093, Mulhouse, France

[b.] Université de Strasbourg, France

[c.] ONET Technologies, 270 chemin des agriculteurs, 26700, Pierrelatte, France

[d.] ONET Technologies, 36 boulevard de l'océan, 13258, Marseille Cedex 9, France

* jean.daou@uha.fr

Table 1 – Summary of the iodine capture performances of TEDA-impregnated activated carbons

Study	Impregnation (wt.%)	Iodine/methyl iodide concentration (I_2, CH_3I)	T (°C)	R.H. (%)	Capture performances
Deuber et al. 1982 [1]	TEDA (nd[1])	1 $mg.m^{-3}$	30	98	P = $5.2*10^{-4}$ %
			130	2	P = $1.8*10^{-3}$ %
Kitani et al. 1972 [2]	HMTA (10 wt.%)	15 ppm (Flow rate : 24 $cm.s^{-1}$)	70	90	P = 29.7 % (E = 70.3 %)
	TEDA (10 wt.%)				P = 9.2 % (E = 90.8 %)
Deuber et al. 1986 [3]	TEDA (nd)	1 $mg.m^{-3}$	130	2	P = $3.7*10^{-3}$ %
			180	<1	P = $1.4*10^{-4}$ %
			130	2	P = $3.4*10^{-4}$ %
			180	<1	P = $5.0*10^{-5}$ %
Deitz et al. 1987 [4]	TEDA (5 wt.%)	nd	30	>90	P = 1.51 ± 0.84 %
Park et al. 1993 [5]	/	0.018 $mol.m^{-3}$	30	nd	Q = 176 $g/kg_{adsorbents}$
		0.460 $mol.m^{-3}$			Q = 775 $g/kg_{adsorbents}$
	TEDA (2.4 %m)	0.011 $mol.m^{-3}$			Q = 179 $g/kg_{adsorbents}$
		0.886 $mol.m^{-3}$			Q = 843 $g/kg_{adsorbents}$
	TEDA (4.9 %m)	0.019 $mol.m^{-3}$			Q = 215 $g/kg_{adsorbents}$
		0.831 $mol.m^{-3}$			Q = 759 $g/kg_{adsorbents}$
Park et al. 2001 [6]	/	0.004 – 0.06 $mol.m^{-3}$	30	Sec	Q = 529 $g/kg_{adsorbents}$
			50		Q = 350 $g/kg_{adsorbents}$
			70		Q = 161 $g/kg_{adsorbents}$
			100		Q = 74 $g/kg_{adsorbents}$
			150		Q = 35 $g/kg_{adsorbents}$
			250		Q = 21 $g/kg_{adsorbents}$
	TEDA (6.5 %m)		30	Sec	Q = 470 $g/kg_{adsorbents}$
			50		Q = 320 $g/kg_{adsorbents}$
			70		Q = 215 $g/kg_{adsorbents}$
			100		Q = 155 $g/kg_{adsorbents}$
			150		Q = 130 $g/kg_{adsorbents}$
			250		Q = 105 $g/kg_{adsorbents}$
González-García et al. 2011 [7]	/	1.75 $g.m^{-3}$	30	95	P = 0.70 % (E = 99.3 %)
	/				P = 27.2 % (E = 72.8 %)
	TEDA (5 wt.%)				P = 1.90 % (E = 98.1 %)
	TEDA (10 wt.%)				P = 3.70 % (E = 96.3 %)
	/				P = 41.4 % (E = 58.6 %)
	TEDA (5 wt.%)				P = 5.90 % (E = 94.1 %)
	TEDA (10 wt.%)				P = 7.70 % (E = 92.3 %)

[1] nd : Not defined

Table 2 – Summary of the iodine capture performances of KI-impregnated activated carbons

Study	Impregnation (wt.%)	Iodine/methyl iodide concentration ($_{I2}$, CH_3I)	T (°C)	R.H. (%)	Capture performances
Kitani et al. 1972 [2]	KI (10 wt.%)	15 ppm, Flow : 24 $cm.s^{-1}$	70	90	P = 6.4 % (E = 93.6 %)
	KI (5 wt.%)	16 ppm, Flow : 24 $cm.s^{-1}$	25	90	P = 2.2 % (E = 97.8 %)
US4016242A 1977 [8]	KIO_3 + KI (0.5 - 4 wt.%) + HMTA (1 wt.%)	5.49 mg iodine in air flow	nd	96	P = 0.16 – 0.50 %
Deitz et al. 1987 [4]	KI	nd[2]	30	95	P = 1.4 ± 0.08 %
	KI + TEDA				P = 0.1 ± 0.84 %
Decourcière 1981 [9]	KI (1 wt.%)	nd	nd	40	DF 10^4
				96	DF < 10^2
Deuber et al. 1982 [1]	KI	1 $mg.m^{-3}$	30	98	P = $6.5.10^{-4}$ %
			130	2	P = $1.5.10^{-4}$ %
Qi-dong et al. 1984 [10]	KI (2 wt.%) + TEDA (2 wt.%)	8 ppmv[3], Flow 28 $cm.s^{-1}$	40	95	DF 10^4
			50		DF $10^{3.5}$
			60		DF 10^3
			70		DF $10^{2.4}$
		8.7 ppmv, Flow 28 $cm.s^{-1}$	40	40	DF 10^4
				50	DF 10^3
				60	DF $10^{2.9}$
Chien et al. 2011 [11]	/	nd	nd	nd	P = 38.7 % (E = 61.3 %)
	/				P = 41.0 % (E = 59 %)
	/				P = 27.7 % (E = 72.3 %)
	/				P = 29.1 % (E = 70.9 %)
	KI (5 wt.%)				P = 0 % (E = 100 %)
Zhou et al. 2014 [12]	KI (>1 wt.%)	nd	≤ 40	≤ 32	P < 0.1 % (E > 99.9 %)<br>DF 10^5

[2] nd : not defined

[3] ppmv : 1 ppmv = 1 µL/L

Table 3 – Summary of the iodine capture performances of silver-exchanged zeolites

Study/Topic	Zeolites (wt.%Ag)	Iodine/methyl iodide concentration (I_2, CH_3I)	Operating conditions	T (°C)	R.H. (%)	Capture performances
Pence et al. 1970 [13] /Cation influence	AgX	CH_3I (0.1 - 10 µg/m^3)	Superficial velocity [4] v = 92 ft/min[5]	25	90	E > 99.9%
	CdX					E = 1.4%
	CuX					E = 1.5 %
	PbX					E = 0.8%
Pence et al. 1970 [13] /Cation influence	NaX	I_2 generated from Dushmann reaction	Superficial velocity v = 46 ft/min	22	90	E = 4.24%
	PbX					E = 7.21%
	TlX					E = 27.37
	AgX		Superficial velocity v = 92 ft/min		100	E > 99.9%
	CuX					E = 0.82
	PbX					E = 5.16
Pence et al. 1970 [13] /Humidity influence	AgX	CH_3I (0.1 - 10 µg/m^3)	Superficial velocity v = 46 ft/min	25	90	E > 99.9%
					95	E > 99.9%
					100	E > 99.9%
			Superficial velocity v = 92 ft/min		90	E = 94%
					95	E = 92%
					100	E = 90%
Evans 1978 [14] /Irradiation influence onto the iodine desorption	AgX	I_2 charged at 6.5 mg/g beforehand	Radiation: 1.5.10^7 rad/h. v = 55 ft/min	35 ≤ T ≤ 80	20 ≤ R.H. ≤ 95	Total penetration P = 0.743 % (E = 99.257) Desorption rate : < 0.1 %
Holladay 1979 [15] /NO_2 poisoning influence	Ag°MOR	1500 mgI_2/m^3	No contaminants	150	nd	Q = 113 g/kg$_{adsorbents}$
			2%NO			Q = 129 g/kg$_{adsorbents}$
			2%NO_2			Q = 68 g/kg$_{adsorbents}$
			2%NO + 2%NO_2 + 2%H_2O			Q = 119 g/kg$_{adsorbents}$
Vance et al. 1982 [16] /Zeolite nature	AgX (37)	nd	nd	130	Dry	Q = 200 g/kg$_{adsorbents}$
	AgY (28)					Q = 210 g/kg$_{adsorbents}$
	AgMOR (20)					Q = 160 g/kg$_{adsorbents}$
Vance et al. 1982 [16] /Temperature influence *AgI stability at high temperature	AgY (28)	nd	nd	130	nd	Q = 210 g/kg$_{adsorbents}$
				600*		Q = 60 g/kg$_{adsorbents}$
				1000*		Q = 50 g/kg$_{adsorbents}$
				1300*		Q = 50 g/kg$_{adsorbents}$
Scheele et al. 1983 [17] /Superficial velocity influence	AgMOR	1.3.10^{-5} mol.L^{-1} CH_3I	Superficial velocity v=3.75 m/min	86	5.10-4 mol.L-1 H2O	Q = 71 g/kg$_{adsorbents}$
				200		Q = 217 g/kg$_{adsorbents}$
			Superficial velocity v=15 m/min	86		Q < 7 g/kg$_{adsorbents}$
				200		Q = 169 g/kg$_{adsorbents}$
Belapurkar et al. 1984 [18] /Temperature influence (Hydrated zeolites. 18%m H_2O)	AgX (16)	7.5 µmol/ml CH_3I	Carrier gas : N_2, 2.5 ml/min	25	nd	Q = 196 g/kg$_{adsorbents}$
				50		Q = 258 g/kg$_{adsorbents}$
				100		Q = 331 g/kg$_{adsorbents}$
				150		Q = 308 g/kg$_{adsorbents}$

[4] Vitesse superficielle : est la vitesse du fluide (gaz) à travers le milieu poreux

[5] ft: unité de longueur US, « the foot ». 1 ft = 0,3048 m.

Table 4 *(continued)*

Study/Topic	Zeolites (wt.%Ag)	Iodine/methyl iodide concentration (I_2, CH_3I)	Operating conditions	T (°C)	R.H. (%)	Capture performances
Belapurkar et al. 1984 [18] /Temperature influence (Dehydrated zeolites)	AgX (16)	7.5 µmol/ml CH_3I	Carrier gas : N_2, 2.5 ml/min	25	nd	Q = 447 $g/kg_{adsorbents}$
				50		Q = 427 $g/kg_{adsorbents}$
				100		Q = 400 $g/kg_{adsorbents}$
				150		Q = 372 $g/kg_{adsorbents}$
Choi et al. 2001 [19] /Silver loading influence	13X (0)	$2.5*10^{-5}$ mol/L CH_3I	4 L/min, v = 0.18 m/s	175	Dry air	Q = 90 $g/kg_{adsorbents}$
	AgX (10)					Q = 170 $g/kg_{adsorbents}$
	AgX (20)					Q = 210 $g/kg_{adsorbents}$
	AgX (30)					Q = 240 $g/kg_{adsorbents}$
Choi et al. 2001 [19] /Temperature influence	AgX (10)	$2.5*10^{-5}$ mol/L CH_3I	4 L/min, v = 0.18 m/s	100	Dry air	Q = 180 $g/kg_{adsorbents}$
				175		Q = 170 $g/kg_{adsorbents}$
				235		Q = 160 $g/kg_{adsorbents}$
				400		Q = 130 $g/kg_{adsorbents}$
Choi et al. 2001 [19] /Silver utilization efficiency	AgX (10)	2.5*10-5 mol/L CH_3I	4 L/min, v = 0.18 m/s	150-250	Dry air	Ag utilization efficiency : 100%
	AgX (20)					Ag utilization efficiency : 85%
	AgX (30)					Ag utilization efficiency : 68%
Choi et al. 2003 [20] /NO_2 poisoning influence	AgX (10)	NO_2 (200 ppm)	4 L/min, v = 0.18 m/s	175	50	E = 99.9%. DF = 10^4
						E = 99%. DF = 10^2 (after 16 months of exposition)
Haefner et al 2010 [21] /Zeolite nature	IONEX Ag-900 (commercial)	33.3 ppm CH_3I	1200 ml/min, 14.1 ft/min, [NO_2] = [NO] = 833 ppm	150	2.5 wt.% H2O	Q = 71.4 $g/kg_{adsorbents}$
	Ag0MOR					Q = 59.5 $g/kg_{adsorbents}$
Soelberg et al. 2012 [22] /Initial I_2 concentration influence	Ag0MOR (16.7)	1.6 ppmv I_2	Carrier gas N_2, [NO_2] = [NO] = 10000 ppm	150	1.5 wt.% H2O	Q = 33 $g/kg_{adsorbents}$
		14.4 ppmv I_2				Q = 80 $g/kg_{adsorbents}$
		49.2 ppmv I_2				Q = 111 $g/kg_{adsorbents}$
Nenoff et al. 2014 [23]	Ag0MOR IONEX Ag-900 (commercial)	57 ppm CH_3I	0.73 L/min, [NO_2] = [NO] = 800 ppm	150	1.8 wt.% H2O	Q = 52 $g/kg_{adsorbents}$ E = 55%
Bruffey et al. 2014 [24]	Ag0MOR (9.5 wt.%) IONEX Ag-900 (commercial)	50 ppm I_2	Superficial velocity v = 10 m/min	150	nd	Q = 72 $g/kg_{adsorbents}$
Bruffey et al. 2014 [24]	Ag0MOR (9.5 wt.%) IONEX Ag-900 (commercial)	50 ppm I_2	Superficial velocity v = 10 m/min	150	nd	Q = 72 $g/kg_{adsorbents}$

Table 5 *(continued)*

Study/Topic	Zeolites (wt.%Ag)	Iodine/methyl iodide concentration (I_2, CH_3I)	Operating conditions	T (°C)	R.H. (%)	Capture performances
Bruffey et al. 2014 [25] /NO_2 poisoning influence	Ag0MOR (9.5 %m) IONEX Ag-900 (commercial)	50 ppm CH_3I	$[NO_2]$ = [NO] = 10000 ppm, v = 4.3 m/min	165	$[H_2O]$ = 6000 ppm	Q = 50 $g/kg_{adsorbents}$
			$[NO_2]$ = [NO] = 10000 ppm, v = 4.3 m/min		$[H_2O]$ < 10 ppm	Q = 56 $g/kg_{adsorbents}$
			$[NO_2]$ = [NO] = 0 ppm, v = 4.3 m/min		$[H_2O]$ < 10 ppm	Q = 125 $g/kg_{adsorbents}$
		50 ppm I_2	v = 10 m/min	150	$[H_2O]$ = 0 ppm	Q = 87 $g/kg_{adsorbents}$
Cheng et al. 2015 [26] / Silver loading influence	13X (0)	2.5 ml/h I_2	Carrier gas: He, 69.3 ml/min, 4mm, sphere	250	Air sec	DF = $10^{2.2}$
				650		DF = $10^{1.1}$
	AgX (10)		Carrier gas: He, 69.3 ml/min, 4mm, sphere	250		DF = $10^{2.7}$
				650		DF = $10^{1.5}$
	AgX (15)		Carrier gas: He, 69.3 ml/min, powder, 40/60 meshes	250		DF = $10^{3.4}$
				650		DF = $10^{3.0}$
	AgX (20)		Carrier gas: He, 69.3 ml/min, powder, 40/60 meshes	250		DF = $10^{3.4}$
				650		DF = $10^{2.9}$
Bruffey et al. 2016 [27]	Ag0MOR (11.9 wt.%) IONEX Ag-900 (commercial)	40 ppb CH_3I	Carrier gas: N_2, v =10 m/min, pellet (d = 16 cm)	150	nd	DF > 190
Nan et al. 2017 [28]	Ag0MOR (12) (commercial)	nd	Pellets (d=1.6mm)	150	nd	E = 88%
Abney et al. 2017 [29]	Ag0MOR (11.9 wt.%) IONEX Ag-900 (commercial)	50 ppmv I_2	Pellets (d = 1.6 mm)	150	Dry air	ND
Azambre et al. 2017[30] Chebbi et al. 2017 [31] / Silver loading influence	AgY (9.1)	1333 ppm	Carrier gas: Ar, 150 mL/min	100	nd	Q_{sat} (mg/g) = 87
	AgY (16.8)					Q_{sat} (mg/g) = 175
	AgY (22.8)					Q_{sat} (mg/g) = 223
	13X (10%m Na)					Q_{sat} (mg/g) = 92
	AgX (7.3)					Q_{sat} (mg/g) = 149
	AgX (23.4)					Q_{sat} (mg/g) = 234
	AgX (35) commercial					Q_{sat} (mg/g) = 267
	AgMOR (5.9)					Q_{sat} (mg/g) = 83
	AgMOR (7.3)					Q_{sat} (mg/g) = 120
	AgMFI (9)					Q_{sat} (mg/g) = 85
	AgFER (4.2)					Q_{sat} (mg/g) = 64
	Ag*BEA (3.4)					Q_{sat} (mg/g) = 46

Notes and references

1 H. Deuber and J.G. Wilhelm, *Proceedings of the 17th DOE Nuclear Air Cleaning Conference, Conf. 820833, U.S. Department of Energy, The Harvard Air Cleaning Laboratory, Denver, Colorado*, 1982, 248–277.

2 S. Kitani, T. Noro, T. Kohara, S. Kitani, T. Noro and T. Kohara, *Journal of Nuclear Science and Technology*, 1971, **9 (4)**, 197–202.

3 H. Deuber, *Nuclear Technology*, 1986, **72**, 44–48.

4 V. R. Deitz, *Carbon*, 1987, **25**, 31–38.

5 S. W. Park, W. K. Lee and H. Moon, *Separations Technology*, 1993, **3**, 133–142.

6 G. Park, I. Kim, J. K. Lee, S. K. Ryu and J. H. Kim, *Science*, 2001, **2**, 9–14.

7 C. M. González-García, J. F. González and S. Román, *Fuel Processing Technology*, 2011, **92**, 247–252.

8 V. R. Deitz and C. H. Blachly, *US4016242A*, 1977.

9 P. Decourcière, *Iodine removal from gaseous effluents in the nuclear industry*, 1981.

10 I. Qi-Dong and H. Sui-yuang, *Proceedings of the 18th DOE Nuclear Airborne Waste Management and Air Cleaning Conference, Conf. 840806, U.S. Department of Energy, The Harvard Air Cleaning Laboratory, Baltimore, Maryland*, 1984, 78–92.

11 C. C. Chien, Y. P. Huang, W. C. Wang, J. H. Chao and Y. Y. Wei, *Clean - Soil, Air, Water*, 2011, **39**, 103–108.

12 J. Zhou, S. Hao, L. Gao and Y. Zhang, *Annals of Nuclear Energy*, 2014, **72**, 237–241.

13 D. T. Pence, F. A. Duce and W. J. Maeck, *Proceedings of the 11th AEC Nuclear Air Cleaning Conference, Conf. 700816, The Harvard Air Cleaning Laboratory, Richland, WA,* 1970, 581–599.

14 A. G. Evans, *Proceedings of the 15th DOE Nuclear Air Cleaning Conference, Conf. 780819, 1, The Harvard Air Cleaning Laboratory, Cambridge, Massachusetts*, 1978, 416–427.

15 D. W. Holladay, *ORNL/ TM-6350, Oak Ridge National Laboratory, Oak Ridge, TN,* 1979.

16 E. R. Vance and D. K. Agrawal, *Journal of Materials Science*, 1982, **17**, 1889–1894.

17 R. D. Scheele, L. L. Burger and C. L. Matsuzaki, *PNL-4489, Pacific Northwest Laboratory, Richland, Washington*, 1983.

18 A. D. Belapurkar, K. A. Rao, N. M. Gupta and R. M. Iyer, *Surface Technology*, 1984, **21**, 263–272.

19 B. S. Choi, G. Il Park, J. H. Kim, J. W. Lee and S. K. Ryu, *Adsorption*, 2001, **7**, 91–103.

20 B. S. Choi, G. Il Park, J. W. Lee, H. Y. Yang and S. K. Ryu, *Journal of Radioanalytical and Nuclear Chemistry*, 2003, **256**, 19–26.

21 D. R. Haefner and T. L. Watson, *Summary of FY 2010 Iodine Capture Studies at the INL, Idaho National Laboratory, Idaho Falls, ID*, 2010.

22 N. Soelberg and T. Watson, *Iodine Sorbent Performance in FY 2012 Deep Bed Tests*, 2012.

23 T. M. Nenoff, M. A. Rodriguez, N. R. Soelberg and K. W. Chapman, *Microporous and Mesoporous Materials*, 2014, **200**, 297–303.

24 S. H. Bruffey, R. T. Jubin, K. K. Anderson and J. F. Walker, *Fuel Cycle Research & Development, Oak Ridge National Laboratory, 30 April , 2014, FCRD-SWF-2014-000465, ORNL/LTR-2014/153*.

25 S. H. Bruffey and R. T. Jubin, *Fuel Cycle Research and Development, Oak Ridge national laboratory, 30 September, 2014, FCRD-SWF-2014-000280, ORNL/LRT-2014/482*, 2014.

26 Q. Cheng, W. Yang, Z. Li, Q. Zhu, T. Chu, D. He and C. Fang, *Journal of Radioanalytical and Nuclear Chemistry*, 2015, **303**, 1883–1889.

27 S. H. Bruffey, R. T. Jubin and J. A. Jordan, *Procedia Chemistry*, 2016, **21**, 293–299.

28 Y. Nan, L. Tavlarides and D. W. DePaoli, *AlChE Journal*, 2017, **63**, 1024–1035.

29 C. W. Abney, Y. Nan and L. L. Tavlarides, *Industrial and Engineering Chemistry Research*, 2017, **56**, 4837–4846.

30 B. Azambre and M. Chebbi, *ACS Applied Materials and Interfaces*, 2017, **9**, 25194–25203.

31 M. Chebbi, B. Azambre, L. Cantrel, M. Huvé and T. Albiol, *Microporous and Mesoporous Materials*, 2017, **244**, 137–150.
